# A Review of the Antimicrobial Properties of Cyanobacterial Natural Products

**DOI:** 10.3390/molecules28207127

**Published:** 2023-10-17

**Authors:** Ian E. Cock, Matthew J. Cheesman

**Affiliations:** 1 Centre for Planetary Health and Food Security, Griffith University, Brisbane, QLD 4111, Australia; 2 School of Pharmacy and Medical Sciences, Griffith University, Southport, QLD 4222, Australia; m.cheesman@griffith.edu.au

**Keywords:** cyanotoxins, cyclic lipopeptides, depsiptide, microcystin, lectins, antibacterial, antimicrobial

## Abstract

The development of multiple-drug-resistant pathogens has prompted medical research toward the development of new and effective antimicrobial therapies. Much research into novel antibiotics has focused on bacterial and fungal compounds, and on chemical modification of existing compounds to increase their efficacy or reactivate their antimicrobial properties. In contrast, cyanobacteria have been relatively overlooked for antibiotic discovery, and much more work is required. This may be because some cyanobacterial species produce environmental toxins, leading to concerns about the safety of cyanobacterial compounds in therapy. Despite this, several cyanobacterial-derived compounds have been identified with noteworthy inhibitory activity against bacterial, fungal and protozoal growth, as well as viral replication. Additionally, many of these compounds have relatively low toxicity and are therefore relevant targets for drug development. Of particular note, several linear and heterocyclic peptides and depsipeptides with potent activity and good safety indexes have been identified and are undergoing development as antimicrobial chemotherapies. However, substantial further studies are required to identify and screen the myriad other cyanobacterial-derived compounds to evaluate their therapeutic potential. This study reviews the known phytochemistry of cyanobacteria, and where relevant, the effects of those compounds against bacterial, fungal, protozoal and viral pathogens, with the aim of highlighting gaps in the literature and focusing future studies in this field.

## 1. Introduction

Cyanobacteria (commonly known as blue-green algae) are photosynthetic prokaryotes that are amongst the oldest oxidative photosynthetic organisms. They have existed for approximately 3.5 billion years, and over that time they have contributed to the Earth’s atmospheric oxygen production [1]. They live in a diverse range of environments, including freshwater, marine environments, soil and bare rock surfaces. They tolerate wide pH, temperature, light and salinity ranges [2]. Cyanobacteria also exist in a range of morphologies, including unicellular (suspended/benthic and aggregate forms) and filamentous forms [3]. Whilst individual cyanobacteria are microscopic, many species are readily visible in nature as they form extensive colonies that form surface crusts or blooms in their environment.

Many cyanobacteria readily switch metabolic pathways between oxygenic and anoxygenic photosynthesis (using sulphide as the electron donor) depending on the environmental conditions [3]. Under normal atmospheric oxygen conditions, all cyanobacteria preferentially use oxygenic photosynthesis for their energy metabolism. However, some species may switch to anoxygenic photosynthesis in the dark or in low-oxygen environments. This metabolic adaptability allows cyanobacteria to survive and flourish in diverse and relatively harsh conditions. The ecological and metabolic variability of cyanobacteria results in their ability to produce a myriad of bioactive compounds, many of which are yet to be rigorously examined for their biotechnology applications [4]. Furthermore, their relatively rapid growth rate compared to higher organisms such as plants and their ability to grow on otherwise non-productive land or in brackish and saline water, industrial wastewater or freshwater make them attractive targets for the production of useful secondary metabolites.

Several reviews have summarised the potential of cyanobacteria as a resource for biofuel and biofertiliser production, and for bioremediation purposes [2,4] and the reader is referred to those reviews for a comprehensive examination of the potential of cyanobacteria for those purposes. Similarly, the nutritional value of cyanobacteria has also been summarised elsewhere [2]. Whilst the therapeutic properties of cyanobacteria and cyanobacterial compounds have also been previously reviewed, most studies have narrow focuses, with the pharmaceutical potential of filamentous marine cyanobacteria being particularly well reported [5]. Furthermore, many reviews into the therapeutic potential of cyanobacteria place substantially greater emphasis on the antioxidant and anticancer properties of cyanobacterial compounds than on other bioactivities [2,4,5,6]. This is perhaps not surprising due to the diversity of cytotoxic compounds identified in cyanobacteria [7]. Whilst environmental production of these toxins is often hazardous, they also have potential in treating cancers. However, the toxicity of some cyanobacterial compounds may also mitigate their usefulness for other therapeutic purposes. For this reason, this review begins by examining some noteworthy toxic cyanobacterial compounds, as they may also have antimicrobial activities. Whilst several reviews have also examined the antimicrobial properties of cyanobacterial compounds [2,4,5,6,8], these activities are often reviewed less comprehensively, or the reviews preferentially focus on the antibacterial and/or antifungal properties of these compounds, with less extensive reporting of their antiprotozoal and antiviral activities. Our study updates the earlier reviews and summarises and extends the antimicrobial properties of cyanobacterial compounds against all classes of infective agents (bacteria, fungi, protozoa, viruses).

## 2. Toxicology

Multiple cyanobacteria spp. secrete toxins into their environment, which are believed to be used as protective mechanisms against competing bacteria, fungi, zooplankton and eukaryotic microalgae. These toxins are particularly harmful to zooplankton that feed on cyanobacteria, directly killing the plankton as well as inhibiting their reproduction [9]. These toxins also cause substantial ecological damage and are threats to wildlife, livestock and human health [10]. Indeed, a recent study reviewed 468 separate reports of cyanobacterial toxin poisoning in humans and animals (including 337 reported incidences since 2000) [11]. The vast majority of these poisonings were due to *Microcystis* spp. (~60% of cases reported in that study), *Anabaena* spp. (~36%), *Aphanizomenon* spp. (~9%), *Planktothrix* spp. (~9%) and *Oscillatoria* spp. (~7%). Notably, the incidence of toxic cyanobacterial blooms has increased rapidly in recent years and has been linked to both human factors (e.g., increased input of nutrients from agricultural fertilisers into water courses) as well as to global climate change events (increased temperatures, increased atmospheric carbon dioxide levels, increased UV intensity) [10]. The increasing threat posed by toxic cyanobacteria blooms requires increased environmental vigilance and improved water quality monitoring. However, it is important to note that not all cyanobacteria blooms produce significant levels of toxins and there are wide discrepancies in opinions, with estimates of toxic blooms ranging from 25 to 75% of total cyanobacterial blooms (as reviewed in [10]).

**Figure 1:**
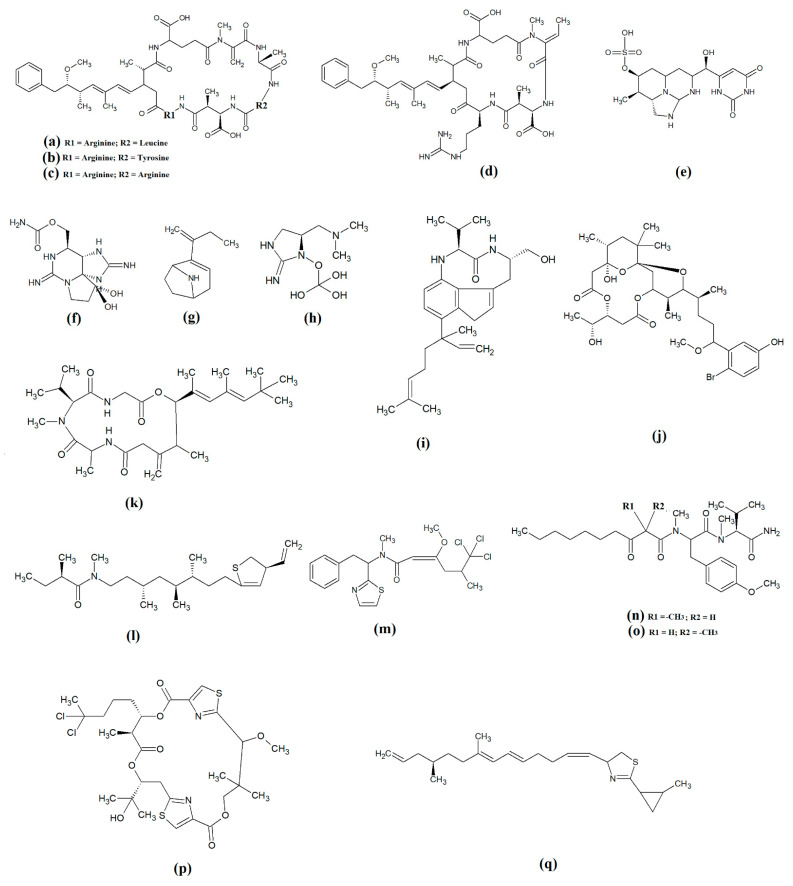
Noteworthy cyanobacterial toxins: (**a**) microcystin-LR; (**b**) microcystin-YR; (**c**) microcystin-RR; (**d**) nodularin; (**e**) cylinderspermopsin; (**f**) saxitoxin; (**g**) anatoxin-a; (**h**) guanotoxin; (**i**) lyngbyatoxin; (**j**) aplysiatoxin; (**k**) antillatoxin; (**l**) kalkitoxin; (**m**) barbamide; (**n**) majuscalamide A; (**o**) majuscalamide B; (**p**) hectochlorin; (**q**) curacin A.

The most common cyanobacterial toxins are the cyclic peptide microcystin compounds, particularly microcystins LR (Figure 1a), YR (Figure 1b) and RR (Figure 1c). These are produced mainly by freshwater *Microcystis* spp., particularly *Microcystis aeruginosa* [10,12]. However, several other freshwater cyanobacterial species, including some from the *Planktothrix*, *Anabaena*, *Oscillatoria*, *Spirulina*, *Synechococcus* and *Trichodesmium* genera, also produce microcystins. Approximately 250 microcystins have been reported [13]. All share similar heptapeptide structures, differing by the specific amino acid moieties in their peptide loops. Microcystins are hepatotoxic and function by covalently binding to the protein phosphatases PP1 and PP2A [7]. Nodularins (Figure 1d), which are produced by *Nodularia* spp. (particularly *Nodularia spumigena*), share similar cyclic peptide structures with the microcystins and also target protein phosphatase enzymes. 

Cylinderospermopsins (Figure 1e) are relatively common cyanotoxins that are mainly produced by freshwater cyanobacteria of the *Cylinderospermopsis*, *Anabaena*, *Aphanizomen*, *Dolichospermum*, *Lyngbya* and *Umezakia* genera [7]. Whilst they are also hepatotoxins, they have very different structures and toxic mechanisms to the cyclic peptide cyanotoxins. Structurally, the cylinderospermopsins consist of a tricyclic guanidine group, linked to a uracil ring moiety. The structure is zwitterionic, making it highly water-soluble. Cylinderospermopsins function by inhibiting protein synthesis [14] and modifying DNA and RNA structure in hepatocytes [15,16]. In addition to their acute toxicity, cylinderospermopsins may also be carcinogenic and have been reported to initiate tumour formation in mice [17], although similar effects are yet to be verified in humans.

Saxitoxins (Figure 1f) are highly potent neurotoxic alkaloids that are best known as shellfish toxins due to their paralytic effects in people that have consumed toxic cyanobacteria of the *Anabaena*, *Aphanizomenon*, *Cuspidothrix*, *Cylinderospermopsis*, *Doliospermum*, *Fischerella* and *Geitlerinema* genera [7]. Approximately 60 saxitoxins have been reported, all of which consist of tricyclic 3,4-perhydropurine systems containing two guanidinium moieties [18]. All saxitoxins are highly toxic and exert their effects by selectively and reversibly blocking voltage-gated sodium channels in neuronal cell synapses, thereby blocking neurotransmission [7].

Anatoxin-a (Figure 1g) is also known as very fast death factor (VFDF) due to its rapid neurotoxic effects. It is produced by multiple species of the *Anabaena*, *Aphanizomenon*, *Arthrospira*, *Cuspidothrix*, *Cylinderospermum*, *Dolichospermum*, *Oscillatoria* and *Phormidium* genera [7]. Structurally, anatoxin-a contains 2-acetyl-9-aza-bicyclo(4.2.1)non-2-ene, which binds to nicotinic acetylcholine receptors and functions as a receptor agonist [19]. Guanotoxin (previously known as anatoxin-a(s); Figure 1g) has only been reported to be produced by *Anabaena* spp. Its structure has been determined to be (5*S*)-2-amino-1-((hydroxylmethoxyphosphinyl) oxy)-*N*,*N*-dimethyl-4,5-dihydri-1H-imidazole-5-methanamine (Figure 1h) [7]. Like anatoxin-a, guanotoxin also inhibits neurotransmission, although this is achieved via inhibition of the enzyme acetylcholinesterase [7,20].

Lyngbyatoxins (Figure 1i) are dermatoxic alkaloids that are produced by several *Lyngbya*, *Oscillatoria* and *Schizothrix* species, particularly *Lynbya majuscula*. To date, seven lyngbyatoxins have been reported [7]. Structurally, all are based on indolactam ring structures, with linayl side chains attached at C-7 [21]. Lyngbyatoxin exposure activates protein kinase enzymes, inducing inflammation and skin blisters [22]. Aplysiatoxins (Figure 1j) have substantially different structures, consisting of 3,4-dihydroxyvaleric acid and 4,6,6,10,12-pentamethyl-3,7,9,11,15-tetraoxy-15-phenylpentadecanoic acid bislactones. Despite these structural differences, aplysiatoxins have similar dermatoxic effects to the lyngbyatoxins [7]. 

Seven lipoprotein cyanotoxins have also been identified. Antillatoxins (Figure 1k) are predominantly produced by the marine cyanobacteria *Lyngbya majuscula* [23]. They consist of a tripeptide moiety that is linked via both ester and amide linkages with the lipid portion of the molecule [24]. Antillatoxins induce neurotoxic effects via activation of voltage-gated sodium channels [23]. Kalkitoxin (Figure 1l) has a structure consisting of 2,4-disubstituted thiazoline linked to a lipophilic chain [25]. It exerts neurotoxic effects in humans by blocking voltage-gated sodium channels. It also strongly suppresses cell proliferation by blocking cell division and it inhibits inflammation. *Lyngbya majuscula* also produces barbamide (Figure 1m), which has strong molluscicidal activity, although no risks to humans have yet been reported [7]. Additionally, *Lyngbya majuscula* produces two majuscalamide cyanotoxins (majuscalamide A and B; Figure 1n and Figure 1o, respectively), which are epimers of *N*-((2*R*)-2-methyl-3-oxodecanoyl)-d-*N*,*O*-dimethyltyrosyl-l-*N*-methylvalinamide [7]. These compounds have therapeutic potential as they have antifungal activity, as well as selective cytotoxicity towards PANC-1 pancreatic and U251N glioblastoma cell lines [26]. *Lyngbya majuscula* also produces hectochlorin (Figure 1p), which has antifungal activity towards *Candida albicans* [27]. That study also reported that hectochlorin modulates actin polymerization and therefore has potent cytotoxic activity against multiple cancer cell lines. Additionally, *Lyngbya majuscula* also produces curacin A (Figure 1q), which exerts potent cytotoxic effects by binding to the colchicine binding site on tubulin, thereby blocking microtubule formation and cell division.

## 3. Cyanobacterial Chemistry

In addition to the potent cyanotoxins discussed above, cyanobacteria also produce a wealth of interesting compounds with therapeutic potential. Perhaps because of the well-established cytotoxicity of some cyanobacterial compounds, the greatest emphasis on cyanobacterial drug research to date has focused on compounds with anticancer activity. However, with the diversity of cyanobacterial compounds already identified, other therapeutic properties should not be neglected. In particular, cyanobacteria contain a wealth of peptides and lipopeptides (both linear and cyclic), as well as lectins, terpenoids and polyphenolic compounds, some of which are summarised below. Whilst the majority of the available chemical literature focusses on marine cyanobacteria, we review the known components from marine, freshwater and soil cyanobacteria herein. 

### 3.1. Linear Peptides and Lipopeptides

Cyanobacteria are a rich source of multiple classes of linear peptides and lipopeptides. Whilst species growing in all environments produce these compounds, the peptide and lipopeptide composition of marine cyanobacteria have been particularly well reported [28]. Therefore, this review has a greater emphasis on linear peptides and lipopeptides identified in marine cyanobacteria. The marine cyanobacterium *Lyngbya majuscula* has been particularly well studied and has yielded a wealth of bioactive structurally diverse secondary metabolites. Whilst our review emphasises the identification of marine cyanobacterial compounds, similar compounds are also produced by freshwater and terrestrial cyanobacterial species, and these are also discussed where relevant in the following sections.

**Figure 2:**
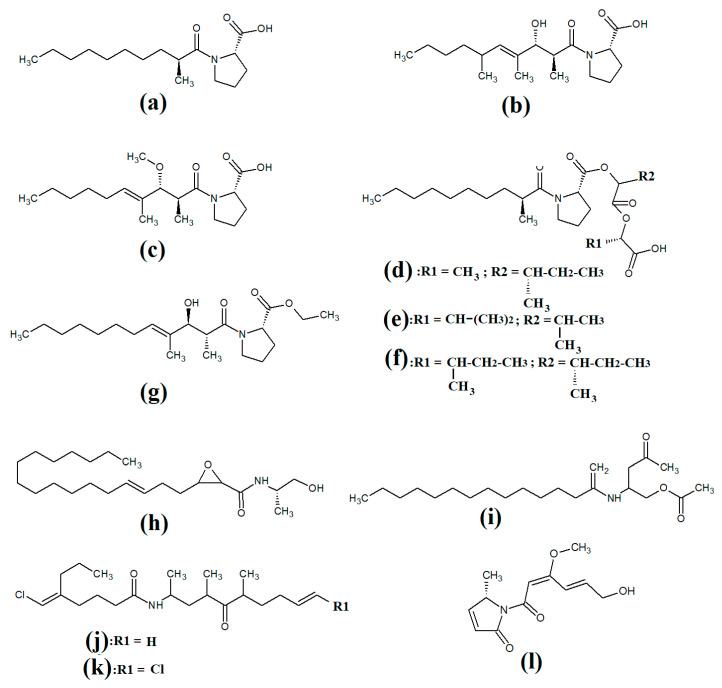
Cyanobacterial tumonoic acid-, besarhananmide-, grenadamine- and palmyrroline-class linear lipopeptides: (**a**) tumonic acid D; (**b**) tumonic acid E; (**c**) tumonic acid F; (**d**) tumonic acid G; (**e**) tumonic acid H, (**f**) tumonic acid I; (**g**) ethyl tumonoate A; (**h**) besarhanamide A, (**i**) besarhanamide B; (**j**) grenadamine B; (**k**) grenadamine C; (**l**) palmyrrolinone.

Tumonoic acid and its derivatives are some of the most abundant linear peptides in many cyanobacteria. Three tumonoic acids (A–C) were initially identified in the marine cyanobacterial species *Lyngbya majuscula* and *Schizothrix calcicola* in 1999 [29]. Since that time, tumonic acid D (Figure 2a), tumonic acid E (Figure 2b), tumonic acid F (Figure 2c), tumonic acid G (Figure 2d), tumonic acid H (Figure 2e) and tumonic acid I (Figure 2f) were identified in *Blennothrix cantharidomum* [30]. Because the tumonoic acids share structural characteristics with homoserine lactones (which function as bacterial signalling molecules), the authors of that study tested their ability to inhibit quorum sensing in *Vibrio harveyi*. Inhibitory activity was reported for all of the tumonoic acids, although tumonoic acid F was particularly potent, with an IC_50_ of 62 μM. That study also reported that tumonoic acid I had noteworthy antimalarial activity (IC_50_ = 2 μM). Subsequently, the tumonoic acid derivative ethyl tumonoate A (Figure 2g) was identified in *Oscillatoria margaritefera* and shown to have anti-inflammatory activity against RAW264.7 murine macrophages in a nitric oxide inhibition assay (IC_50_ = 9.8 μM) [31]. Besarhanamide A (Figure 2h) and besarhanamide B (Figure 2i) were also isolated from *Lyngbya majuscula* extracts and their structures were reported [32]. The therapeutic potential of the besarhanamides remain to be rigorously explored, although the authors of that study reported that they had moderate toxicity in an *Artemia* nauplii lethality assay.

In another study, grenadamide B (Figure 2j) and grenadamide C were isolated from lipophilic *Lyngbya majuscula* extracts [33]. Both compounds have planar fatty acid amide structures. Grenadamide C (Figure 2k) was determined to have a substituted vinyl chloride group. The same study also reported that both of these compounds have weak insecticidal properties against armyworms, with mortality rates 38–50% at 1 mg/mL. Other biological activities remain relatively unexplored for these compounds and further studies are required. 

**Figure 3:**
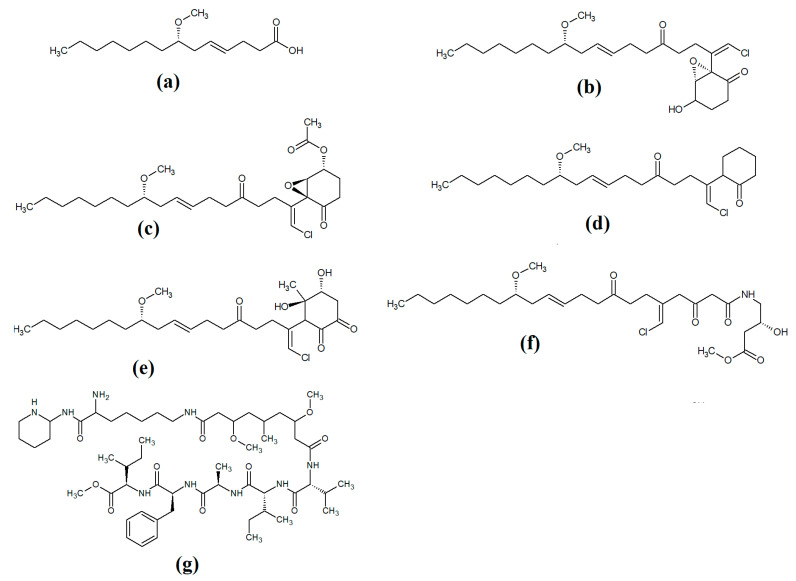
Cyanobacterial malyngamide-class linear lipopeptides: (**a**) lyngbic acid; (**b**) 8-*epi*-malyngamide C; (**c**) 8-*O*-acetyl-8-*epi*-malyngamide C; (**d**) isomalyngamide K; (**e**) malyngamide 2; (**f**) malyngamide 3; (**g**) mitosoamide A.

Lipopeptides of the malyngamide class are also common across multiple cyanobacteria of the genus *Lyngbya*. Indeed, more than 30 malyngamide class lipopeptides have been reported in multiple cyanobacterial species, with the majority of these identified in *Lyngbya majuscula* [34,35]. Whilst these compounds vary considerably, all contain a 7*S*-methoxytetradec-4(*E*)-enoic acid (commonly known as lyngbic fatty acid; Figure 3a) chain, or a lyngbic acid derivative. Several of the malyngamides have been reported to have noteworthy bioactivities. In particular, the malyngamide stereoisomers 8-epi-malyngamide C (Figure 3b) and 8-*O*-acetyl-8-epi-malyngamide C (Figure 3c) are strongly cytotoxic against NCI-H460, Neuro-2a and HT29 cells, with IC_50_ values ranging from 3.1 to 23.9 μM [34,35]. Additionally, 8-epi-malyngamide C also inhibits bacterial quorum sensing in transformed *Escherichia coli* [35]. Isomalyngamide K (Figure 3d) was isolated from *Lyngbya majuscula* extracts in another study, although no bioactivities were reported for this compound [36] and further studies are required to evaluate its therapeutic potential.

Another study isolated malyngamide 2 (Figure 3e) from *Lyngbya sordida* and determined that it contains a trihydroxy cyclohexanone ring structure [37]. The authors reported noteworthy nitric oxide inhibitory activity against LPS-stimulated RAW264.7 murine macrophages (IC_50_ = 8 μM). They also reported that malyngamide 2 had moderate cytotoxic activity against H-460 human lung cancer cells (IC_50_ = 8 μM). However, the antimicrobial properties of this compound are yet to be rigorously evaluated. Another study reported the isolation and identification of malyngamide 3 (Figure 3f) from *Lyngbya majuscula* and reported that it has weak cytotoxicity against MCF-7 breast cancer cells (IC_50_ = 29 μM) and HT-29 colon cancer cells (IC_50_ = 48 μM) [38]. Additionally, mitsoamide A (Figure 3g) was isolated from a marine cyanobacterium of the *Geitlerinema* genus and was reported to have several unusual structural features [39]. The structure contains a homolysine, 3,7-dimethoxy-5-methyl-nonanedioic acid and a piperidine moiety, linked by an alanine, isoleucine, N-methyl-isoleucine, phenylalanine and valine peptide. The authors of that study reported that mitsoamide A was cytotoxic to human NCI-H460 lung cells (IC_50_ = 0.46 μM).

**Figure 4:**
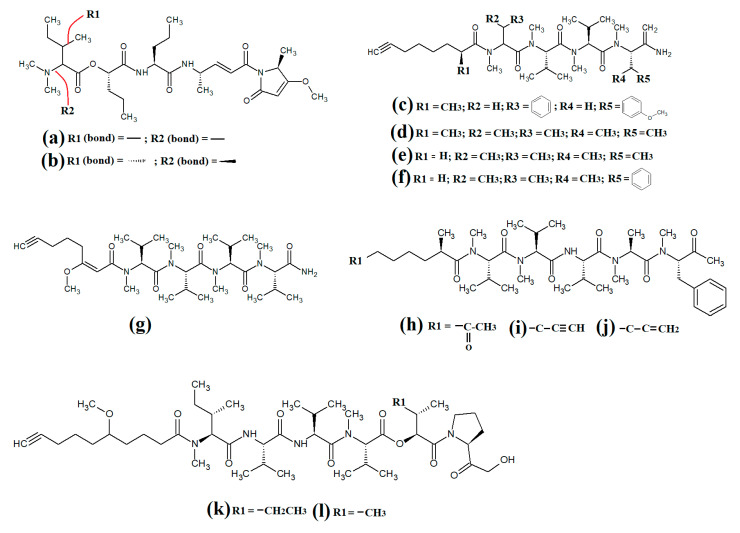
Cyanobacterial gallinamide-, symplostatin- and dragonamide-class linear lipopeptides: (**a**) gallinamide A; (**b**) symplostatin 4; (**c**) dragomabin; (**d**) dragomabin B; (**e**) dragomabin D; (**f**) dragomabin E; (**g**) dragomabin C; (**h**) almiramide A; (**i**) almiramide B; (**j**) almiramide C; (**k**) viridamide A; (**l**) viridamide B.

The lipodepsipeptide gallinamide A (Figure 4a) was isolated from *Schizithrix* spp., although the specific species was not identified [40]. Interestingly, gallinamide A had moderate antiprotozoal activity against *Plasmodium falciparum* (IC_50_ = 8.4 μM) and *Leishmania donovani* (IC_50_ = 9.3 μM). The same study also reported moderate cytotoxicity against human NCI-H460 lung cancer cells and neuro-2a mouse neuroblastoma cells. A different study isolated and identified the structural isomer symplostatin 4 (Figure 4b) from *Symploca* spp., although the specific species was not identified [41]. The authors of that study reported that symplostatin 4 had noteworthy cytotoxicity against HT-29 human colon cancer cells (IC_50_ = 53 μM) and HeLa cervical cancer cells (IC_50_ = 12 μM) due to its antimitotic activity. Symplostatin 4 disrupted intracellular microtubule formation at 50 μM, and completely depolymerised microtubules at 100 μM. Additionally, the authors also reported that symplostatin 4 and largazole (which was also produced by the *Symploca* spp. cells) synergised the cytotoxicity of the combination against HT-29 cells, although fractional IC_50_ values of the combination components were not reported. Largazole functions via a different cytotoxic mechanism, through inhibition of histone deacetylases [42], possibly accounting for these effects.

Dragomabin (Figure 4c) and the dragonamides B (Figure 4d), D (Figure 4e), E (Figure 4f) and C (Figure 4g), as well as the almiramides A (Figure 4h), B (Figure 4i) and C (Figure 4j) and viridamides A (Figure 4k) and B (Figure 4l), are a group of structurally related linear lipopeptides. All contain an eight-carbon polyketide moiety linked to amino acids via amide bonds, with all of these compounds except the viridamides terminating in a primary amide functional group [7]. Dragomabin and dragonamides A and B were isolated from *Lyngbya majuscula* and screened against a chloroquine-resistant *Plasmodium falciparum* strain [43]. The authors reported IC_50_ values between 4.3 and 7.7 μM for all compounds except dragonamide B, which was completely inactive against the chloroquine-resistant *Plasmodium falciparum* strain, indicating that the aromatic moiety is required for antimalarial activity. Additionally, the same study also determined the cytotoxicity of these compounds against Vero cells, with IC_50_ values ≤ 182 μM for all of the tested compounds. Dragonamides C and D were isolated from *Lyngbya majuscula* and *Lyngbya polychroa* in another study and reported to have noteworthy inhibitory activity against *Leishmania donovani* (IC_50_ = 5.1 μM) [44] and U2OS osteocarcoma cells (IC_50_ = 56–59 μM).

Almiramides A–C were isolated from *Lyngbya majuscula*, identified and screened for antiprotozoal activity against *Leishmania donovani* [44]. The inhibitory activity of almiramides B and C were particularly noteworthy (IC_50_ values of 2.4 and 1.9 μM, respectively). Another study screened the almiramides against a chloroquine-resistant *Plasmodium falciparum* strain and reported good antimalarial activity for dramonamide A (IC50 = 7.7 μM) [45]. Another study isolated viridamide A (Figure 4k) and viridamide B (Figure 4l) from the marine cyanobacteria *Oscillatoria nigro-viridis* and determined that the structures consisted of N-methylated amino acids, hydroxyl acids and a 5-methoxydec-9-ynoic acid moiety [46]. The authors of that study reported noteworthy antiparasitic activity for viridamide A against *Leishmania mexicana*, *Plasmodium falciparum* and *Trypanosoma cruzi* (IC_50_ values 1.1–5.8 μM). Contrastingly, the effects of all of these compounds against bacteria, fungi, viruses and other protozoal pathogens are yet to be rigorously examined. 

**Figure 5:**
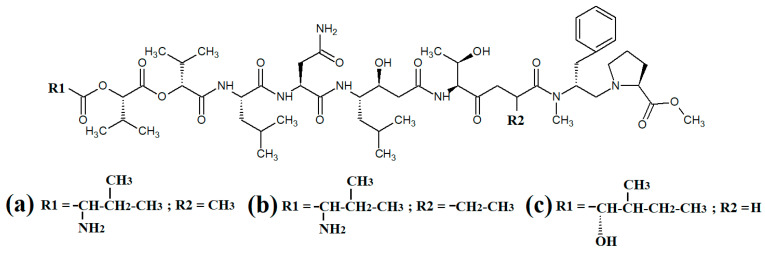
Cyanobacterial grassystatin linear lipopeptides: (**a**) grassystatin A; (**b**) grassystatin B; (**c**) grassystatin C.

Grassystatins are linear decadapsipeptides that are potent inhibitors of cathepsin E enzyme [5]. Grassystatins A (Figure 5a), B (Figure 5b) and C (Figure 5c) were isolated and identified from the marine cyanobacterium *Lyngbya conferoides* [47]. That study reported that these compounds are potent inhibitors of both cathepsin D (IC_50_ = 16.9 nM) and E (IC_50_ = 0.62 nM). As cathepsins D and E are involved in antigen processing via the HHC class II pathway and bioactive protein degradation [48], it is likely that grassystatins may block pathogen activation mechanisms, and thereby modulate the course of multiple infections. Notably, grassystatins have profound effects on the course of viral diseases. By inhibiting cathepsin enzymes, grassystatins block viral attachment glycoprotein activation and therefore inhibit the entry of virus into the target cells, as well as directly inhibiting viral replication [49].

### 3.2. Linear Lipopeptides and Peptides Containing Heterocyclic Moieties

Lyngbyapeptin D (Figure 5a) and three structural analogues (27-deoxylyngbyabellin A, lyngbyabellin J and laingolide B) have been identified in *Lyngbya boullonii* extracts [50]. That study reported that all of these compounds were moderately cytotoxic towards HT29 colorectal adenocarcinoma and HeLa cervical cancer cell lines, and therefore may have potential as cancer therapeutics. Notably, the effects of lyngbyapeptin D and its analogues have yet to be tested against bacterial, fungal, protozoal and viral pathogens and substantially more work is required.

**Figure 6:**
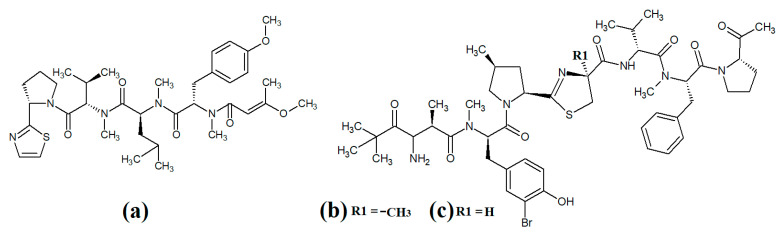
Cyanobacterial lyngbypeptin- and bisebromoamide-class heterocycle containing linear lipopeptides: (**a**) lyngbyapeptin D; (**b**) bisebromoamide; and (**c**) norbisebromoamide.

The cytotoxic linear peptide bisebromoamide (Figure 6b) was isolated from an unidentified *Lyngbya* spp. in 2009 [51]. A subsequent study also identified the structural analog norbisebromamide (Figure 6c) [52]. Bisebromoamide is highly cytotoxic against an extensive panel of human cancer cells with an average GI_50_ value of 40 nM [53]. That study reported that bisebromoamide exerts its anticancer effects via modulation of extracellular signal-regulated protein kinase (ERK) pathways. Another study reported that bisebromoamide also destabilises actin filaments [54]. However, the effects of bisebromoamide and norbisebromoamide are yet to be elucidated.

### 3.3. Cyclic Depsipeptides and Peptides

Cyclic depsipeptides are a diverse group of compounds containing ring structures composed of amino and hydroxyl acid moieties linked by amide and ester bonds. Differences in the ring structure and the nature of the side chains provides substantial diversity in the structure and function of this class of compounds [55].

### 3.4. Cyclic Depsipeptides Containing Heterocyclic Moieties

**Figure 7:**
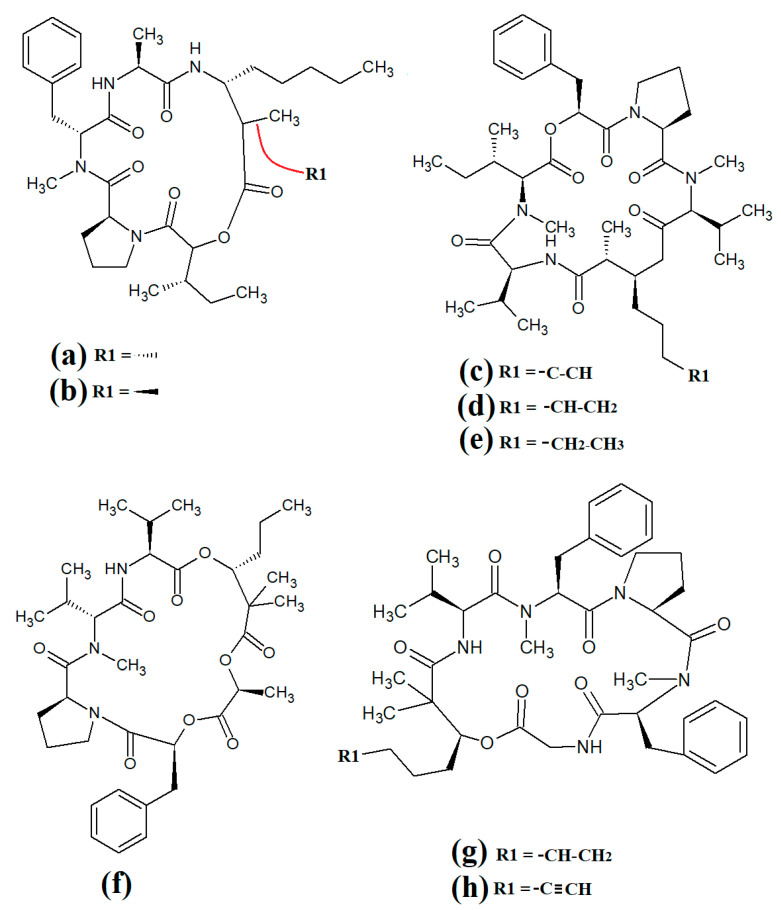
Cyclic cyanobacterial lipopeptides: (**a**) porpoisamide A; (**b**) porpoisamide B; (**c**) hantupeptin A; (**d**) hantupeptin B; (**e**) hantupeptin C; (**f**) palmyramide A; (**g**) cocosamide A; (**h**) cocosamide B.

The epimeric cyclic depsipeptides porpoisamide A (Figure 7a) and porpoisamide B (Figure 7b) were isolated from an unidentified marine *Lyngbya* spp. cyanobacterium and the structure was elucidated [56]. Both compounds contained alanine, N-methyl-phenylalanine, 2-hydroxy-3-methylpentanoic acid and 3-amino-2-methyl-octanoic acid moieties and differed only in the stereochemistry of the amino acid at C2 of the ring structure. That study reported that both porpoisamides have weak cytotoxic activity against human HCT-116 colorectal (IC_50_ = 21 μM) and U2OS osteosarcoma cell lines (IC_50_ = 28 μM).

Hantupeptin A (Figure 7c) and its structural analogues hantupeptin B (Figure 7d) and hantupeptin C (Figure 7e) have been isolated from *Lyngbya majuscula* extracts [57]. These compounds each contain a cyclic structure consisting of four amino acids and two hydroxy acid residues, one of which is a PKS-derived 3-hydroxy-2-methyloctynoic acid. The hantupeptins differ only in the nature of a chain extension that occurs at C2 of this residue. Subsequent studies by the same group reported significant cytotoxic activity against MOLT-4 leukaemia and MCF-7 breast cancer cell lines, with IC_50_ values between 32 and 3000 nM [58]. Hantupeptin A was particularly potent against MOLT-4 cells (32 nM). However, the potential of these compounds in the treatment and inhibition of pathogenic diseases remains to be examined.

The 19-membered cyclodepsipeptide palmyramide A (Figure 7f) has been reported in *Lyngbya majuscula* extracts [59]. Its structure was reported to consist of five amino acid moieties, including three valine residues, as well as one residue each of *N*-methyl-valine and proline. It also contains the three hydroxy acids 2,2-dimethyl-3-hydroxyhexanoic acid, lactic acid and 3-phenyllactic acid. The authors of that study tested palmyramide A in sodium channel blocking assays and reported noteworthy inhibition of veratridine and ouabain-induced sodium channel overload, resulting in considerable cytotoxicity in neuro-2a murine neuroblast cells (IC_50_~17 μM) and in H-460 human lung cancer cells (IC_50_~40 μM).

The cyclodepsipeptides cocosamide A (Figure 7g) and cocosamide B (Figure 7h) have also been isolated from *Lyngbya majuscula* extracts in another study [38]. Both compounds contain two *N*-methyl-phenylalanine residues, proline, glycine, valine, and either 2,2-dimethyl-3-hydroxy-7-octynoic acid or 2,2-dimethyl-3-hydroxy-7-octenoic acid. Both compounds were moderately cytotoxic against HT-29 and MCF-7 breast cancer cells, with IC_50_ values ranging from 11 to 39 μM. The effects of these compounds against bacterial, fungal, protozoal and viral pathogens remain to be investigated.

**Figure 8:**
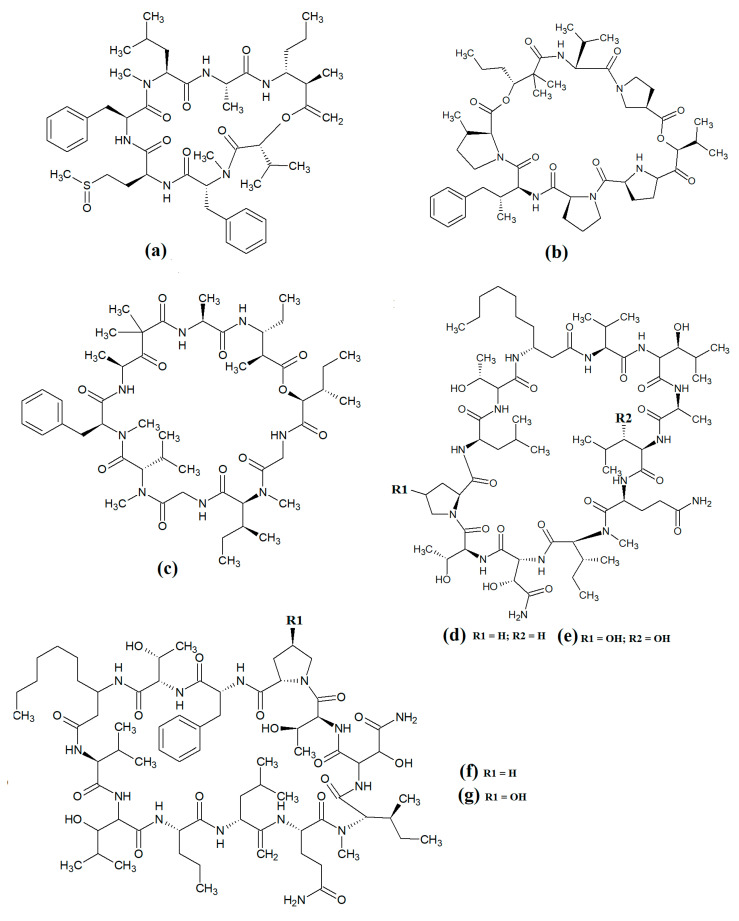
Cyclic cyanobacterial lipopeptides: (**a**) carriebowmide; (**b**) pitiprolamide; (**c**) desmethoxymajusculamide C; (**d**) laxaphycin B2; (**e**) laxaphycin B3; (**f**) lynbyacyclamide A; (**g**) lynbyacyclamide B.

Carriebowmide (Figure 8a) was isolated from *Lyngbya majuscula* extracts and the structure was determined to consist of a 21-membered cyclic depsipeptide structure, which consists of alanine, *N*-methyl-leucine, phenylalanine, methionine, *N*-methyl-phrnylanine and a 2-hydroxy-3-methylbutyric acid moiety [60]. It also contains the two rare amino acids 3-amino-2-methyl-hexanoic acid and methionine sulfoxide. Another study reported that carriebowmide is moderately cytotoxic towards human HEK-293 embryonic kidney cells, with an IC_50_ of approximately 50 μM [33]. However, we are unaware of any studies to date that have screened carriebowmide against any pathogens and substantially more work is needed to evaluate the therapeutic potential of this compound.

Pitiprolamide (Figure 8b) was also identified in *Lyngbya majuscula* extracts [61]. Its structure was reported to be similar to that of dolastatin, although it contains four proline residues, as well as a valine, dolaphenvaline and 2-hydroxy-isovaleric acid moieties. The authors of that study reported that pitiprolamide was mildly cytotoxic towards human MVC7 breast cancer and HCT116 colorectal cancer cell lines (IC_50_ = 33 μM for both). Of further interest, pitiprolamide also showed antibacterial activity against *Mycobacterium tuberculosis* in a disc diffusion assay at 50 μg. However, the MIC was not determined, making it impossible to compare with the potency of other compounds tested in other studies. Additionally, the disc diffusion assay was perhaps not the most appropriate assay system to test antibacterial activity of this compound. Pitiprolamide has a molecular mass of 905.54 Da and its size would hinder its diffusion in agar gels, thereby providing falsely low results. Furthermore, dolastatin (which is structurally similar) has low water solubility, also hampering its diffusion in agar. Therefore, liquid-based assay systems may have been more appropriate for testing the antibacterial potency of this compound. Despite that, the reported mycobacterial activity is promising, and future studies should focus on testing pitiprolamide against other pathogens using more appropriate assays.

Another cyclic depsipeptide was isolated from the marine cyanobacterium *Lyngbya majuscula* and was identified as desmethoxymajusculamide C (Figure 8c) [62]. Notably, desmethoxymajusculamide C has highly potent and selective cytotoxicity, with an IC_50_ value of 20 nM against human HCT-116 colorectal cells, although it is substantially less potent against other cell lines. The authors of that study determined the HCT-116 cytotoxicity to be mediated through inhibition of microfilament production. 

Laxaphycin B2 (Figure 8d) and laxaphycin B3 (Figure 8e) were detected in *Annabaena torulosa* extracts, albeit in low abundance [63]. Both compounds were reported to have noteworthy anti-proliferative activity towards a panel of drug-resistant and drug-sensitive solid lymphoblastic cancer cells, with IC_50_ values generally between 1 and 20 μM. Interestingly, these compounds substantially potentiated each other’s anti-proliferative activity when tested in combination, with at least a two-fold increase in potency against all of the cell lines tested. Lynbyacyclamide A (Figure 8f) and lynbyacyclamide B (Figure 8g) are structurally related to the laxaphycins, which were originally isolated from *Lyngbya majuscula* extracts [64]. Structurally, they contain 37-member rings consisting of 11 α-amino acids and 1 β-amino acid (β-amino-decanoic acid). Similar to laxaphycin B2 and laxaphycin B3, the lynbyacyclamides are strongly cytotoxic, both with IC_50_ values of 0.7 μM against mouse B16 melanoma cancer cells. Interestingly, whilst the cytotoxic properties of the laxaphycin and lynbyacyclamides are well established, we were unable to find reports testing their activities against any pathogens and substantial further work is required.

**Figure 9:**
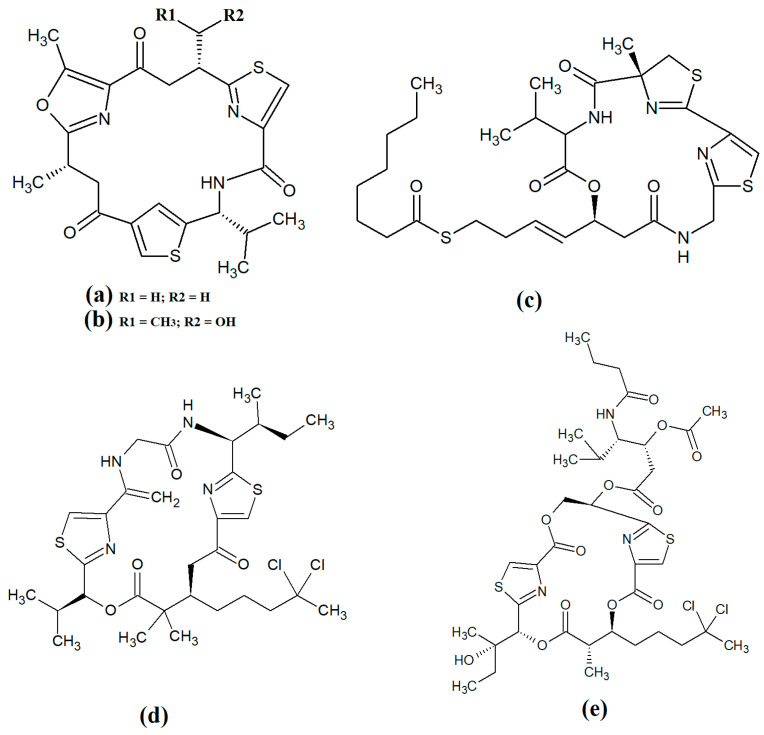
Cyclic cyanobacterial depsipeptides/peptides: (**a**) ventutramide A; (**b**) ventutramide B; (**c**) largazole; (**d**) 27-deoxylyngbyaybellin A; (**e**) lyngbyabellin J.

Several recent studies have focused on cyanobacterial compounds for antiprotozoal activity, particularly against malaria-, leishmaniasis- and schistomiasis-causing pathogen species. Several promising compounds have been identified with noteworthy antiprotozoal activities. In particular, ventutramide A (Figure 9a) and ventutramide B (Figure 9b) that were isolated from *Oscillatoria* spp. are reported to have good antimalarial activity. Ventutramide A was a particularly good inhibitor of *Plasmodium falciparium* growth, with an IC_50_ of 8.2 μM [65]. Both compounds also inhibited *Trypanasoma cruzi* and *Leishmania donovani*, albeit with substantially higher IC_50_ values. The ventutramides are structurally unusual, containing two thiazole and one methyl-oxazole ring moieties, which may contribute to the antiprotozoal activity of these compounds. The effects of these compounds on non-protozoal pathogens have been less extensively examined and substantially more work is required to determine the therapeutic potential of these molecules. 

Largazole (Figure 9c) was initially isolated from marine cyanobacteria of the genus *Symploca* in 2008 [42]. This compound has attracted substantial interest since that time as it is a potent class I histone deacetylase (HDAC) inhibitor. As HDACs regulate HIV latency [66], largazole has potential as an anti-retroviral therapy for use in HIV-AIDS. Additionally, the HDAC inhibitory activity of largazole also makes it a promising target for anticancer drug development. Notably, largazole (as well as its analogues) has anti-proliferative activity towards the NCI 60 panel of human cancer cell lines [67]. Further studies demonstrated that largazole also has cytostatic effects in a human HCT116 xenograft mouse model via stimulation of histone hyperacetylation, as well cytotoxic effects by inducing apoptosis [67]. Notably, these compounds are yet to be tested for anti-infective properties against pathogens of interest to human health. 

Lyngbyabellins are a group of cyclic lipopeptides that are characterised by the presence of thiazole/thaizoline moieties and dichlorination of a polyketide moiety. The structural lyngbyabellin analogues 27-deoxylyngbyaybellin A (Figure 9d) and lyngbyabellin J (Figure 9e) were isolated and characterised from *Lyngbya bouillonii* extracts and tested for cytotoxic activity against human HT29 colorectal and HeLa cervical carcinoma cell lines [50]. Whilst both compounds displayed good cytotoxic activity, 27-deoxylyngbyaybellin A was particularly potent, with IC_50_ values of 12 and 7.3 nM against the HT29 and HeLa cells, respectively. Lyngbyabellin J also had noteworthy activity, albeit with significantly higher IC_50_ values (54 and 41 nM, respectively). 

**Figure 10:**
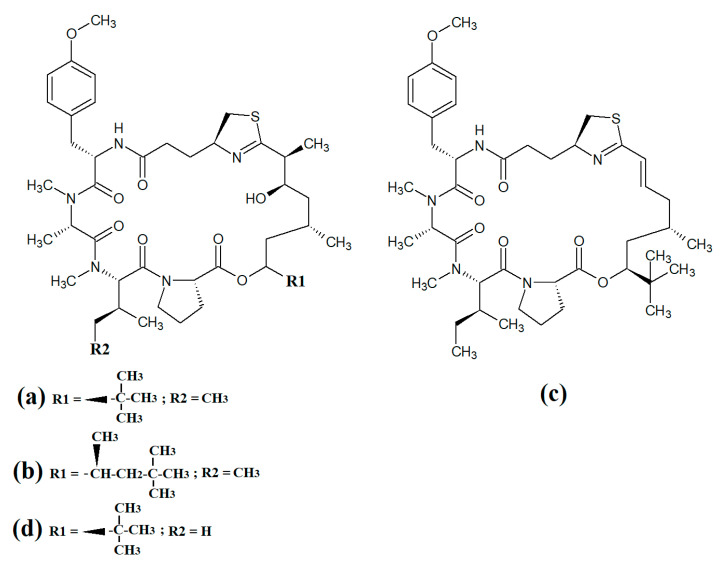
Cyclic cyanobacterial apratoxins: (**a**) apratoxin A; (**b**) apratoxin D; (**c**) apratoxin E; (**d**) apratoxin G.

Multiple members of the apratoxin class of compounds have also been identified in cyanobacteria. These compounds have cyclic structures that contain a thiazoline unit, as well as an extensive polyketide moiety. Apratoxin A (Figure 10a) was reported over twenty years ago in the marine cyanobacterium *Lyngbya majuscula* [68]. Since that time, apratoxin D (Figure 10b), apratoxin E (Figure 10c) and apratoxin G (Figure 10d) have also been isolated from *Lyngbya majuscula*, as well as from *Lyngbya sordida* [69]. Apratoxin D was reported to have potent cytotoxic activity against human H-460 lung cancer cells (IC_50_ = 2.6 nM) [69]. Apratoxin E also has potent cytotoxicity against HT29, HeLa and U2OS cell lines (IC_50_s of 21, 72 and 59 nM, respectively) [70]. Similarly, apratoxin G was cytotoxic to human H-460 cells (IC_50_ = 14 nM) [71]. However, these compounds are yet to be rigorously tested against pathogens.

**Figure 11:**
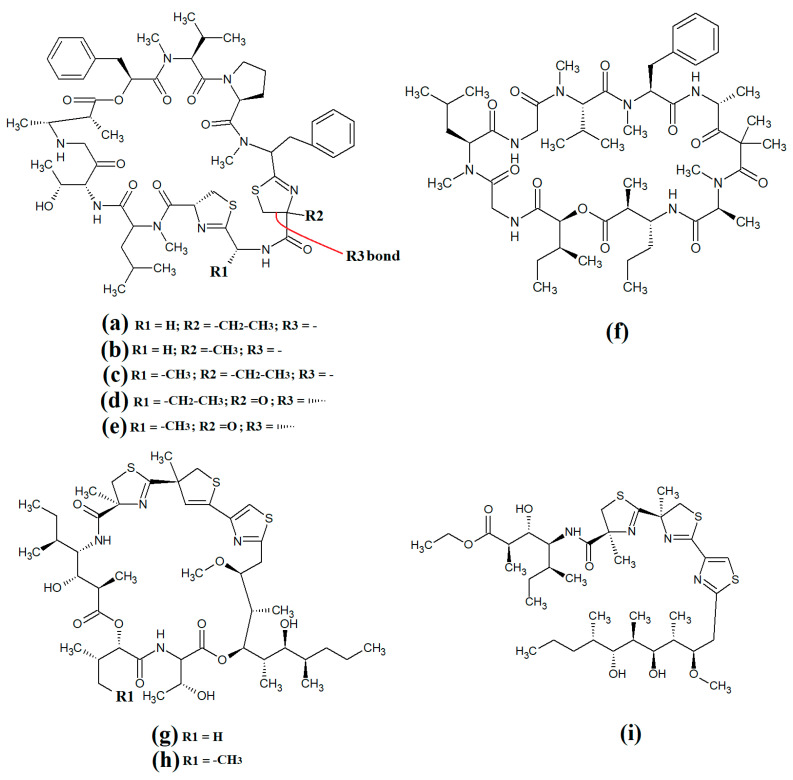
Cyanobacterial grassypeptolide- and hoiamide-class depsipeptides/peptides: (**a**) grassypeptolide A; (**b**) grassypeptolide B; (**c**) grassypeptolide D; (**d**) grassypeptolide F; (**e**) grassypeptolide G; (**f**) ibu-epidemethoxylyngbyastatin; (**g**) hoiamide A; (**h**) hoiamide B; (**i**) hoiamide C.

Grassypeptolide A (Figure 11a) and grassypeptolide B (Figure 11b) were isolated and characterised from *Lyngbya confervoides* [72,73]. The authors of those studies also screened these compounds and reported noteworthy cytotoxic activity for grassypeptolide A against human U2OS osteosarcoma, HeLa cervical cancer, HT29 colorectal and IMR-32 neuroblastoma cells, with IC_50_ values of 2.2, 1, 1.5 and 4.2 μM, respectively [72]. Subsequent studies demonstrated that grassypeptolides A and C inhibit cell cycle progression at G1 phase in low concentrations, and also at the G2/M phase when tested at higher concentrations [73]. More recently, grassypeptolide D (Figure 11c), grassypeptolide F (Figure 11d) and grassypeptolide G (Figure 11e) were isolated from the marine cyanobacteria *Leptolyngbya* spp. and *Lyngbya majuscula* and tested for cytotoxicity [74,75]. Grassypeptolides D and E were particularly cytotoxic against HeLa (IC_50_ = 335 and 192 nM, respectively) and mouse neuro-2a-blastoma cells (IC_50_ = 599 and 407 nM, respectively) [74]. In contrast, grassypeptolides F and G were only moderate inhibitors of transcription factor AP-1 (IC_50_ = 5.2 and 6 μM, respectively) [75].

The hoiamides are a class of cyclic depsipeptides that have been isolated from several filamentous marine cyanobacteria, including *Lyngbya majuscula* and *Phormidium gracile* [76]. Hoiamide A (Figure 11g) contains an extended isoleucine moiety, two methylated thiazoles, a thiazole moiety and a highly methylated and oxygenated polyketide group [76]. The same study also reported that hoiamide A is a potent inhibitor of [3H]-batrachotoxin binding to voltage-gated sodium channels (VGSCs), and therefore increases sodium influx in neural cells (IC_50_ = 93 nM; EC_50_ = 2.3 μM). A subsequent study isolated and identified hoiamide B (Figure 11h) and hoiamide C (Figure 11i) from other marine cyanobacteria [77]. Notably, that study reported that hoiamide B also promotes sodium influx into neocortical neural cells (EC_50_ = 3.9 μM)

### 3.5. Lariat-Type Cyclic Depsipeptides

**Figure 12:**
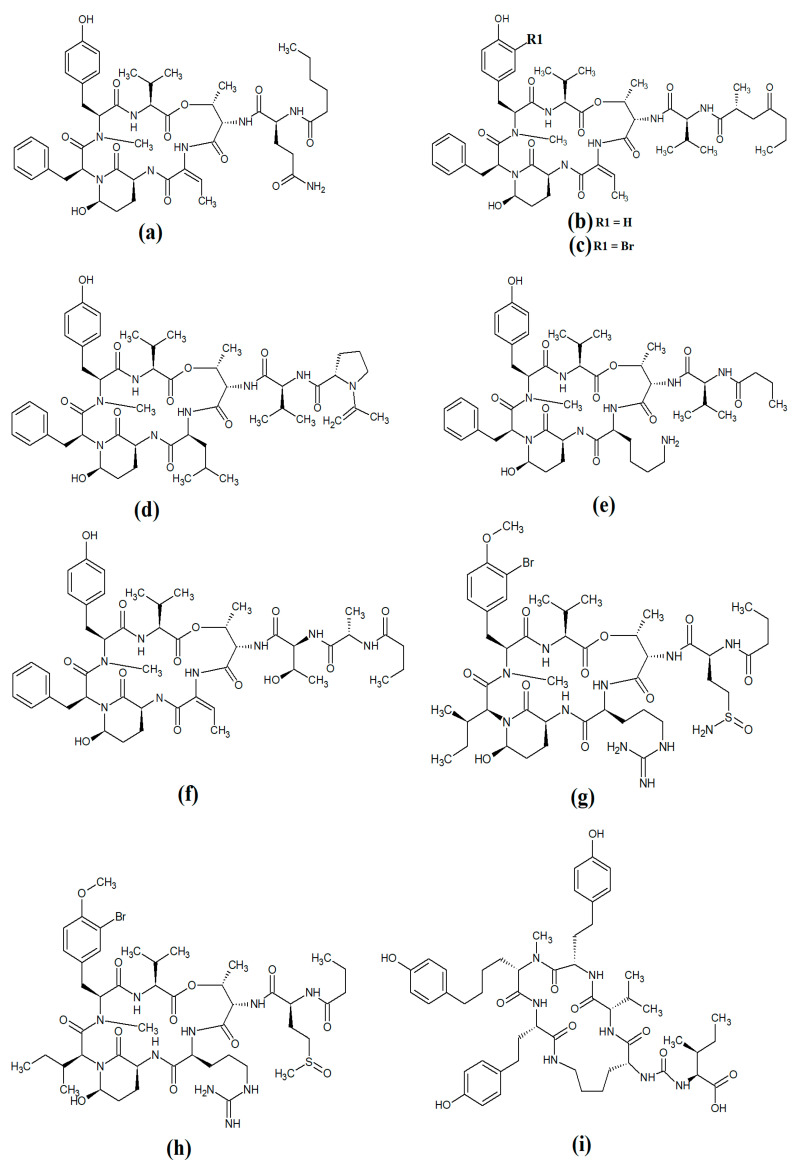
Cyanobacterial lynbyastatins and kempopeptins: (**a**) lynbyastatin 7; (**b**) bouillomide A; (**c**) bouillomide B; (**d**) kempopeptin A; (**e**) kempopeptin B; (**f**) molassamide; (**g**) symplocamide A; (**h**) pompanopeptin A; (**i**) pompanopeptin B.

Lariat-type cyclic depsipeptides are a class of compounds with a wide range of ring sizes and tail lengths. What they all have in common is that they contain non-peptide polyketide regions within their structures [78]. Many of these compounds have relatively low polarity and therefore have good membrane permeability, allowing them to affect intracellular targets. Additionally, many compounds of this class have good serine protease inhibitory activity. The lyngbyastatins are one such class of molecules with noteworthy biological properties. Cyanobacterial-derived lynbyastatins include lynbyastatin 7 (Figure 12a), bouillomide A (Figure 12b), bouillomide B (Figure 12c), kempopeptin A (Figure 12d), kempopeptin B (Figure 12e), molassamide (Figure 12f), symplocamide A (Figure 12g), pompanopeptin A (Figure 12h) and pompanopeptin B (Figure 12i). As serine proteases are involved in multiple important processes in human health, including immunological responses and blood clotting, compounds that modulate serine protease activities may have profound effects on inflammation, cancer, cardiac health, etc. 

Lynbyastatin 7 was originally isolated and characterised from *Lyngbya confervoides* [79]. The authors of that study also reported strong elastase inhibitory activity for lynbyastatin 7 (IC_50_ = 8.3 nM), as well as similar activity for the structural analogues lynbyastatins 5 and 6. In another study, bouillomide A and bouillomide B were isolated from *Lyngbya bouillonii* and their structures were elucidated [80]. These compounds were reported to be selective inhibitors of serine proteases, with substantial inhibitory effects against elastase (IC_50_ = 1.2 μM for both compounds) and chymotrypsin (IC_50_ values of 2.6 and 0.32 μM for bouillomides A and B, respectively). Notably, their inhibitory activity was substantially lower against the other serine proteases tested. 

Kemopopetins A and B were isolated from an unidentified marine cyanobacterium of the genus *Lyngbya* in 2008 [81]. These compounds were noteworthy inhibitors of elastase and chymotrypsin activity, although kemopopetin A had the most potent inhibitory activity (IC_50_ values of 0.32 and 2.6 μM against elastase and chymotrypsin, respectively). In a different study, molassamide was isolated from the cyanobacterium *Dichothrix utahensis*, making it the first natural product identified in any species from the genus *Dichothrix* [82]. Molassamide was also a good inhibitor of elastase and chymotrypsin activity (IC_50_ values of 32 and 234 nM, respectively). 

In another study, symplocamide A was identified in an unidentified marine bacterium of the *Symploca* genus [83]. The structure was unusual amongst this class of compounds as it contained citrilline and N,O-dimethyl-bromo-tyrosine residues within the cyclic backbone structure. The authors of that study reported that symplocamide A was a good inhibitor of chymotrypsin and trypsin protease activity (IC_50_ = 0.38 and 80.2 μM, respectively). Additionally, this compound had potent cytotoxicity towards NCI-H460 human lung cancer cells (IC_50_ = 40 nM) and murine neuro-2a neuroblastoma cells (IC_50_ = 29 nM). Symplocamide A was also a noteworthy inhibitor of *Plasmodium falciparum* growth (IC_50_ = 950 nM).

Pompanopeptins A (Figure 12h) and B (Figure 12i) were isolated and characterised from *Lyngbya confervoides* [84]. Despite their similar names, these compounds are not structurally related, due to the presence of a *N*-methyl-2-amino-6-(4′-hydroxy-phenyl) hexanoic acid moiety in the cyclic backbone of pompanopeptin B. Pompanopeptin A (but not pompanopeptin B) was determined to be a selective inhibitor of trypsin, with an IC_50_ of 2.4 μM. Notably, with the exception of the antiprotozoal activities of some compounds discussed above, the anti-pathogenic properties of all lyngbyastatins have been largely neglected and substantially more work is required to understand their therapeutic potential.

**Figure 13:**
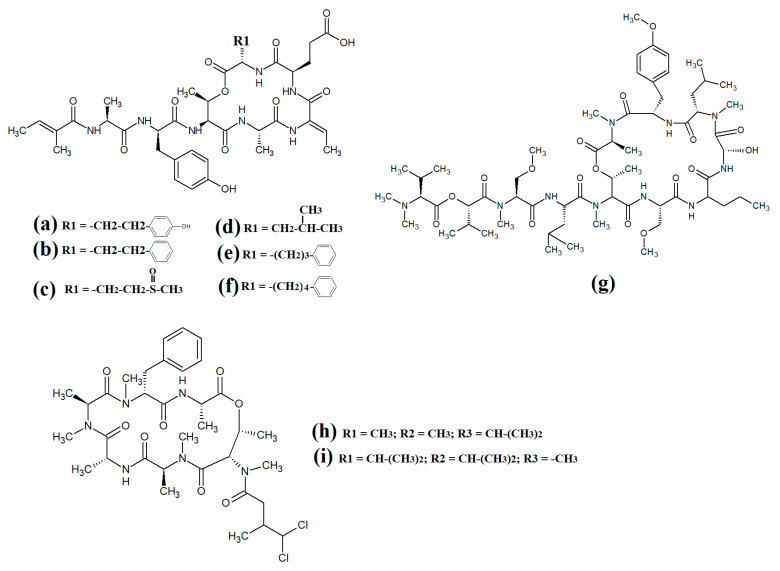
Lariat-type cyanobacterial compounds: (**a**) tiglicamide A; (**b**) tiglicamide B; (**c**) tiglicamide C; (**d**) largamide A; (**e**) largamide B; (**f**) largamide C; (**g**) coibamide A; (**h**) itralamide A; (**i**) itralamide B.

Further lariat-type cyclic depsipeptides have been isolated from *Lyngbya confervoides*. In particular, the structural analogues tiglicamide A (Figure 13a), tiglicamide B (Figure 13b), tiglicamide C (Figure 13c), largamide A (Figure 13d), largamide B (Figure 13e) and largamide C (Figure 13f) were identified and their structures were determined to differ by a single amino acid residue in the cyclic backbone [85]. The authors reported that these compounds were moderate inhibitors of elastase activity, with IC_50_ values ranging from 0.53 to 7.3 μM.

Another study reported the isolation and characterisation of coibamide A (Figure 13g) [86]. The authors reported that the compound was highly *N*-methylated, with eight of the eleven amino acids in the macrocyclic group containing *N*-methyl moieties. Interestingly, coibamide A displayed potent cytotoxicity against human NCI-H460 lung cancer cells and murine neuro-2a cells (LC_50_ values < 23 nM against both cell lines). Additionally, coibamide A was screened against the 60 human cancer cell lines of the NCI panel, and the authors reported substantial toxicity against MDA-MB-231 (IC_50_ = 2.8 nM), LOX IMVI (IC_50_ = 7.4 nM), HL-60(TB) (IC_50_ = 7.4 nM) and SNB-75 cells (IC_50_ = 7.6 nM) [86]. However, we were unable to find studies that screened coibamide A against any pathogens of importance to human health.

Itralamide A (Figure 13h) and itralamide B (Figure 13i) were isolated from *Lyngbya majuscula* and their structures were reported [33]. Interestingly, both compounds contained a branched chlorinated group (4,4-dichloro-3-methylbutanoic acid) linked to N-methyl-threonine. The itralamides are both toxic, although itralamide B was particularly toxic towards human HEK-293 embryonic kidney cells (IC_50_ = 6 μM). 

### 3.6. Cyclic Depsipeptides with Extensive Polyketide Chain

**Figure 14:**
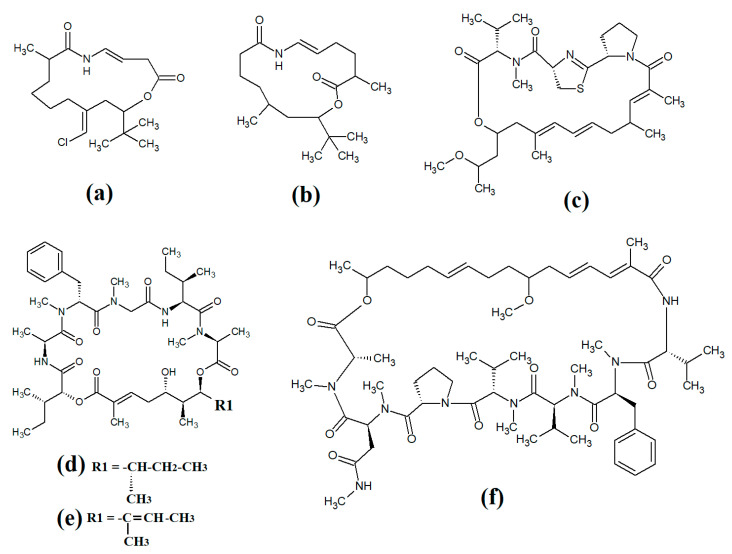
Cyclic cyanobacterial depsipeptides with extensive polyketone chains: (**a**) laingolide B; (**b**) palmyrolide A (**c**) alotamide A; (**d**) lagunamide A; (**e**) lagunamide B; (**f**) malevamide E.

Laingolide B (Figure 14a) was isolated from the marine cyanobacteria *Lyngbya bouillonii* and structurally characterised [50]. Unfortunately, this compound is highly labile and biological activity testing was not undertaken in that study. Palmyrolide (Figure 14b) contains a similar cyclic structure, with extensive polyketone groups, and was isolated from mixed cyanobacterial cultures rich in *Oscillatoria* spp. [87]. Interestingly, palmyrolide A was a moderate inhibitor of calcium influx in cerebrocortical neurons (IC_50_ = 3.7 μM) via blocking of sodium channels (IC_50_ = 5.2 μM). In another study, alotamide A (Figure 14c) was isolated from *Lyngbya bouillonii* and it was determined to consist of a macrocyclic structure containing seven acetate units, as well as N-methylvaline, a thiazoline moiety and a proline, linked by a polyketide group [88]. Interestingly, alotamide A was reported to activate calcium influx in cerebrocortical neurons (IC_50_ = 4.2 μM).

A related class of compounds (lagunamides) were also isolated from *Lyngbya majuscula* extracts and their structures were elucidated [89]. The authors of that study reported that both lagunamide A (Figure 14d) and langunamide B (Figure 14e) were strongly cytotoxic towards murine P338 leukaemia cells (IC_50_ values of 6.4 and 20.5 nM, respectively). These compounds also had significant antimalarial potential, with IC_50_ values of 0.2 and 0.9 μM against *Plasmodium falciparium*. Additionally, lagunamide A and lagunamide B had substantial anti-swarming activity against *Pseudomonas aeruginosa*, although IC_50_ values were not reported in that study, making comparisons difficult. Despite this wide range of promising bioactivities, most pathogens of human importance are yet to be tested using these compounds.

Malevamide E (Figure 14f) is a structural analogue of dolastatin that was isolated from the marine cyanobacterium *Symploca laete-viridis* [90]. This compound is a noteworthy inhibitor of calcium entry into immortalised human HEK embryonic kidney cells treated with thapsigargin. However, the mechanism of inhibition was not reported. We were unable to find studies that tested this compound against any pathogen of medicinal importance in humans. Substantially more work is required. 

## 4. Antimicrobial Properties

### 4.1. Antibacterial Activity

Compounds isolated from cyanobacteria possess inhibitory activities towards multiple bacterial species, as summarised in Table 1. This is interesting given the wide structural dissimilarity between many of the molecules that have been tested. Additionally, many of the experiments were performed on agar, where purified preparations of the molecules produce a zone of inhibition, or in microdilution broth assays where minimum inhibitory concentrations (MICs) can be quantified. Together, these provide evidence of the effectiveness of molecules derived from cyanobacteria in inhibiting the growth of various pathogens.

The abietane diterpenes produced MIC values of 14–22 µg/mL against *S. aureus, S. epidermidis, S. typhi, V. cholerae, B. cereus, B. subtilis, E. coli* and *K. pneumoniae* [91]. Aeruginazole A inhibits *B. subtilis*, *S. aureus* and *S. epidermidis* with IC_50_ values of 2.2 µM, although it did not inhibit *E. coli* [92,93]. Ambigols A–C inhibited *B. megaterium* in agar diffusion assays at a concentration of 100 nM [94,95]. However, in all these studies, the toxicities of the compounds were not determined. Ambiguine isonitriles are potent inhibitors of *B. anthracis*, *M. tuberculosis* and *S. aureus,* where MIC values as low as 78 ng/mL have been observed with a moderate toxicity of 40 µM observed against Vero cells for ambiguine G nitrile [96,97,98]. Anaephenes A, B and C inhibit *S. aureus*, as well as its resistant counterpart, MRSA, in addition to *M. luteus* and *B. cereus*, with low MIC values and moderate toxicity towards HCT116 cells being reported [99,100]. Anyhdrohapaloxindole A possesses moderate-to-strong activities against *M. tuberculosis, M. smegmatis, S. aureus*, *E. coli* and *A. baumannii* but is toxic towards Vero cells [101], while antillatoxin B is a potent inhibitor of *B. cereus* growth but is weaker against *S. typhimurium* and *L. monocytogenes* [102], although toxicity measurements were not conducted in this study.

Bastadin has antibacterial properties, although the affected microbial species were not identified in the relevant study, nor was the activity or toxicity of bastadin quantified [104]. Bromoanaindolone has mild inhibitory activity towards *B. cereus*, with an MIC value of 530 µM [107], while *Mycobacteria* spp. are effectively inhibited by brunsicamines A, B and C [108]. In both these studies, toxicity determinations were not conducted. Both carbamidocyclophane F and G strongly inhibit *Mycobactetium tuberculosis*, with lower activities towards *Acinitobacter baumannii*, *P. aeruginosa*, *S. aureus*, *S. pneumoniae* and *E. faecalis* [111]. Additionally, they are toxic against two different cancer cell lines. Carriebowlinol is an inhibitor of *Vibrio* and *Fusarium* spp., although its toxicity is yet to be assessed [114]. *Bacillus cereus*, *E. coli* and *S. epidermidis* are inhibited by comnostins A–E [115] and are highly cytotoxic towards Caco-2 and KB cells, while low MIC values (4–32 µg/mL) have been reported for cybastacines A and B against *E. faecalis*, *E. faecium*, *M. abscessus*, *N. carnea*, *N*, *cyriacigeorgica*, *S. pyogenes*, *S. aureus* and *S. epidermidis*, although the toxicities of these compounds were not determined [122]. The cylindrofridins A–C and cylindrocyclophanes A–C are also potent inhibitors of *S. aereus*, with MIC values of 1 μg/mL, although the MIC values measured were substantially higher against MRSA, *S. pneumoniae*, *E. faecium* and other bacteria [123]. The compounds were toxic towards the HaCaT keratinocyte cell line. Corioloc acid and dimorphecolic acid are active towards *B. subtilis*, *M. flavus*, *S. aureus* and *S. epidermidis* on agar, although large masses of these compounds (50–100 µg per disc) were used in the assays [124]. Unfortunately, that study did not determine an MIC, making it impossible to evaluate the potency of those compounds, or to benchmark their activity against other antibacterial molecules, and the toxicities of these compounds were not reported. Another study by Choi et al. [116] reported that the crossbyanols (B–D) produce low MIC values against both the susceptible *S. aureus* bacteria as well as the resistant MRSA pathogen, indicating their potential as antibiotic chemotherapies. However, crossbyanol B was toxic in brine shrimp lethality assays, with all compounds showing weak or no activity in lung cancer cell line assays. These different bioactivity profiles provide information on the safety and potential therapeutic usefulness of the four compounds.

*Mycobacterium tuberculosis* growth can be strongly suppressed with eucapsitrione (MIC values ranging from 3.1 to 6.4 µM), although this compound was determined to be considerably weaker against *M. smegmatis*, *S. aureus* and *E. coli*, and has limited cytotoxicity towards Vero cells [127]. Fischerellins A and B generate MIC values of 2–100 µM against *S. aureus*, *M. tuberculosis*, *M. smegmatis* and *E. coli*, whilst fischambiguine B is equally as effective against *S. aureus*, *B. anthracis*, *M. tuberculosis* and *M. smegmatis* [97]. Both molecules show a low level of cytocoxity towards Vero cells. Several different hapalindole compounds are moderately toxic while possessing potent activities towards a wide range of bacteria on agar, including *S. aureus B. subtilis*, *E. coli*, *E. faecium*, *S. epidermidis S. pyogenes*, *S. pneumoniae*, *H. influenza*, *K. pneumoniae*, *P. morganii*, *Salmonella* spp. and *S. sonnei*, producing zones of inhibition (ZOIs) as large as 36 mm and MIC values ranging from 0.5 to 16 µg/mL [129,130]. Kawaguchipeptins A and B mildly inhibit *S. aureus* growth, although their toxicities are unreported [137], while lagunamides A, B and C inhibit the swarming properties of *P. aeruginosa* and show strong cytotoxicity towards multiple cancer cell lines [89,138]. Laxaphycins A, B and B3 are moderately active against *B. cereus*, *S. typhimurium* and *L. monocytogenes* but have not been assessed for their toxicities [102]. Agar disc diffusion assays to evaluate the antibacterial activity of linoleic acid reported varying degrees of activity across the bacterial species *B. subtilis*, *Micrococcus flavus*, *S. aureus* and *S. epidermidis* when 100 µg of each compound was infused in the discs [124]. However, linoleic acid is quite nonpolar and therefore agar diffusion assays are not the best choice of assay to evaluate its activity. ϒ-Linolenic acid is generally a more effective antibacterial compound against *S. aureus*, *E. coli*, *K. aerogenes*, *P. aeruginosa* and *S. typhi* based on their relatively low MIC values of 2–16 μg/mL [142]. The toxicity of these molecules is as yet unknown. Shaala et al. [144] demonstrated that lyngbic acid and the malyngamides (4, A and B) inhibit *M. tuberculosis*, whilst other studies report that the lyngbyazothrins strongly inhibit *B. subtilis* and *E. coli* growth on agar [146,147,148]. Inhibition of B*. cereus*, *S. aureus* and *S. pyogenes* by malyngoloide has been reported but was not quantified, making it difficult to compare their activity to that of other natural products and clinical antibiotics [149]. The toxicity of the lyngbyazothrins is not known, while they have been determined for the other compounds using several different mammalian cell lines.

Majusculamide compounds have moderate antibacterial activity against *S. typhimurium* and *L. monocytogenes*, although they are more potent inhibitors of *B. cereus* [102]. These compounds are yet to be tested for their cytotoxicities. Microcystin-LR is a strong inhibitor of *Mycobacterium* spp. where low MIC values (60 nM–1.93 µM) have been documented against *M. chelonae*, *M. kansaii*, *M. terrae* and *M. tuberculosis* [154]. These findings are particularly promising given the seriousness of mycobacterial infections and their limited treatment options, and also given that they were found to be nontoxic against a hepatoma cell line [154]. Additionally, these compounds should be tested against drug-resistant mycobacterial strains in order to determine their effectiveness against these particularly dangerous organisms. Low levels of activity were observed for muscoride against *B. subtilis* in agar diffusion assays, although its toxicity was not reported [157]. The two compounds 20-nor-3α-acetoxy-abieta-5,7,9,11,13-pentaene and 20-nor-3α-acetoxy-12 hydroxy-abieta-5,7,9,11,13-pentaene were not assessed for their toxicity levels; however, they do appear to have broad-spectrum properties since they can inhibit *S. aureus*, *S. epidermidis*, *S. typhi*, *V. cholerae*, *B. subtilis*, *B. cereus*, *E. coli* and *K. pneumoniae*, with their strongest activity reported against *Staphylococcus* spp. [91]. Norharmane (9*H*-pyrido(3,4-b)indole and 4,4’-dihydroxybiphenol also showed good antibacterial activity (MIC values of 16–160 μg/mL) against *E. coli*, *P. aeruginosa*, *S. aureus*, *B. subtilis* and *B. cereus* [158]. Activity was also reported for noscomin against *B. cereus*, *S. epidermidis* and *E. coli* [159]. These compounds were not examined for their cytotoxicities. *Propionibacterium acnes* growth was also inhibited in agar diffusion when 50 μg of nostocionone, nostocionone D1, nostocionone D2 or nostocionone D3 was infused into discs [161], although their toxicities were not reported in this work.

Potent inhibition of *S. aureus* and *B. subtilis* was observed for the compound nostocyclyne A, with MIC values of 30–36 nM [163] indicating their potential for therapeutic use, although their toxicities still need to be measured. Low MICs (2–16 μg/mL) were also reported for nostotrebin 7 and nostolactone 7 against several bacteria, including *E. faecium*, *B. subtilis*, *S. aureus*, *M. tuberculosis*, *E. aerogenes*, *S. typhi*, *P. aeruginosa* and *E. coli* [129,164], while toxicity studies for these molecules are lacking. Pahayokolides A and B are acutely toxic in zebrafish assays However, they also inhibited the growth of *B. subtilis*, *M. megaterium*, *P. aeruginosa*, *M. luteus*, *E. coli* and *S. epidermidis*, with substantially stronger inhibition noted for *Bacillus* spp., with 5 μg/mL of these compounds individually producing ZOIs as large as 32 mm [167,168]. The potencies of these compounds were not determined, and the use of agar diffusion methods may not be a suitable measure of activity due to the large sizes of these molecules and thus their lowered capacity to move through the agar medium. C-Phycocyanin also inhibits *B. subtilis*, although it is less effective against *Pseudomonas* and *Xanthamonus* spp. [169]. This study also showed that this compound is nontoxic in a silkworm assay. Parsiguine has inhibitory properties towards *S. epidermidis*, while its toxicity is undetermined [170]. Phycocyanin, which was isolated from three different cyanobacterial species, showed good antibacterial activity on agar towards *S. aureus*, *E. coli* and *Pseudomonas* and *Klebsiella* spp. when using 4–100 µg of the compound and, in microdilution broth assays, yielded MIC values of 50–125 µg/mL against these bacteria [171,173]. Future studies on this compound should include an examination of its cytotoxicities. The pitipeptolides A and B are strong inhibitors of *M. tuberculosis* in agar diffusion assays, although MIC values were not determined in that study and only weak cytotoxicity was observed against Vero cells [174]. Similarly, pitiprolamide inhibited *B. cereus* growth in disc diffusion assays with a low cytotoxicity [175]. Falch and colleagues [94] reported antibacterial activity against *M. luteus*, *B. subtilis* and *E. coli* by protoamides, although the potency was not determined in the study and toxicity studies were not performed. Additionally, schizotrin A was shown to produce large ZOIs on agar at concentrations of 7 nM against *B. subtilis* in another early study [177] but again, toxicity assays were not conducted.

The compound scytoscalarol was shown to be weakly cytotoxic in a Vero cell assay and it exerted inhibitory activity towards *B. anthracis*, *S. aureus*, *E. coli* and *M. tuberculosis*, with MIC values varying between 2 and 110 µM [184]. Three tiahuramide molecules (A, B and C) are inhibitors of *S. baltica*, *A. salmonicida*, *V. anguillarum*, *M. luteus* and *E. coli* and have anticancer properties against neuroblastoma cells [192]. Falch et al. [193] state that they were able to demonstrate inhibition of *M. luteus*, *B. subtilis* and *E. coli* by tjipanazole D, but did not quantify the inhibition nor did they assess the toxicity of the compound.

### 4.2. Antifungal Activity

Considerable evidence has accumulated on the inhibitor effects of compounds isolated from cyanobacteria with antifungal properties. *Candida albicans* has been particularly well studied and is susceptible to growth inhibition by several cyanobacterial molecules. Scytonema sp. produce scytoscalarol, a sesterterpenene containing a guanidino group, which was isolated by bioactivity-guided fractionation. This compound was shown via microdilution broth assays to inhibit *C. albicans*, with an MIC of 4 µM [184]. Thecyanobacteria *Fischerella ambigua* also contains indole alkaloids, including ambiguine isonitriles, which have similar potency towards *C. albicans* [98]. Additionally, halopindane-related alkaloids isolated from *Fischerella ambigua* have noteworthy antifungal activity against *C. albicans*. Fischambiguine A and ambiguine P were particularly good inhibitors of *C. albicans* growth, with MIC values in the range 15–30 µM [97]. Both *Westiellopsis* sp. and *Fischerella muscicola* contain anhydrohapaloxindole A, which strongly inhibits the growth of *C. albicans*, with an MIC of 1.9 µM [101]. *Calothrix fusca* contains the cyclic decapeptide calophycin, which inhibits *C. albicans* on agar at 1.2 µg/disc and in broth at an MIC value of 1.25 µg/mL [109]. An environmental *Nostoc* spp. strain was found to contain carbamidocyclophane compounds that are active against *C. albicans*, with some producing MIC values ranging from 1 to 10 µM [111]. Other compounds showing activity towards *C. albicans* include lyngbyabellin B from the marine cyanobacterium *Lyngbya majuscula* ([145], tanikolide from *Scytonema* spp. [190,191] and the cyclic peptides tolybyssidins A and B, which were isolated from *Tolypothrix byssoides* [194]. Norharmane (9H-pyrido(3,4-b)indole) is a cyanobacterial β-carboline exometabolite from *Nodularia harveyana* and *Nostoc insulare* that possesses noteworthy inhibitory activities in microdilution broth assays towards *C. albicans*, with the biphenyl compound 4,4’-dihydroxybiphenol being a slightly more potent growth inhibitor [158]. However, an anthraquinone derivative, eucapsitrione, isolated from *Eucapsis* spp., was inactive against *C. albicans* when tested at a concentration of 55 µM [127].

Many compounds purified from cyanobacteria inhibit the growth of other fungal strains and/or show broad-spectrum antifungal properties. For example, *Anabaena cylindrica* contains the balticidins A–D which exert potent and specific antifungal activity against *Candida maltosa* on agar, with ZOIs of 3–12 mm at 10 µg [103]. However, that study did not determine MIC values, making comparisons with other studies difficult. *Fischerella ambigua* isolated from soil samples in Iran was found to contain a novel compound named parsiguine, which inhibited *Candida krusei* with an MIC value of 20 µg/mL [170]. Methanolic extracts prepared from the terrestrial blue-green alga, *Nostoc commune*, contains a lipopeptide nostofungicidine that has potent antifungal activity against *Aspergillus candidus* in microdilution broth assays with an MIC value of 1.6 µg/mL [160]. The allelochemical compounds fischerellins A and B identified in *Ficscherella muscicola* elicit full inhibition of the agronomically important fungal strains *Uromyces appendiculatus* and *Erysiphe graminis* at 250 ppm and 1000 ppm, respectively [128]. The authors of that study only tested these two relatively high concentrations of these compounds to allow comparisons with other compounds and other studies. The cyanobacterium *Hassallia* sp. contains hassallidins A and B, which possess MIC activities ranging from 4 to 16 µM against a number of different fungal strains, including *C. albicans*, *C. krusei*, *C. glabrata*, *C. tropicalis*, *C. parapsilosis*, *C. neoformans* and *A. fumigatus* [131,132]. Scytophytins (isolated from *Scytonema* spp.) and tolytoxins (isolated from *Tolypothrix* spp.) are further examples of cyanobacterial compounds with potent broad-spectrum antifungal activity, with ZOIs of up to 30 mm being observed on agar against the fungal strains *S. pastorianus*, *N. crassa*, *C. albicans*, *P. ultimum*, *R. solani* and *S. homoeocarpa* [178,179,180,181,182,183]. Whilst these ZOIs indicate potent activity, the compounds were tested at a single relatively high dose and MICs were not reported. As previously mentioned, calophycin purified from *Calothrix fusca* inhibits *C. albicans*; however, it is also has broad-spectrum capability in that it also inhibits *P. notatum*, *Aspergillus oryzae*, *Saccharomyces cerevisae* and *Trichophyton mentagrophytes* on agar and in broth at potencies that are comparable to the positive control antifungal drug amphotericin B [109]. The carmaphycins A and B, isolated from *Symploca* blue-green algae species, produced IC_50_ values of 2.5 and 2.6 nM against the *S. cerevisiae* 20S proteasome and was shown to be cytotoxic to colon and lung cancer cell lines with IC_50_ values of 6–43 µM [195].

Some compounds isolated from cyanobacteria act synergistically in inhibiting fungal growth. Ethanolic extracts of the blue-green algae *Anabaena laxa* contain laxaphycins, which have antifungal inhibitory activities on agar against *C. albicans*, *A. oryzae* and *S. cerevisiae* [139,140,141]. The activities of laxaphycins A–E vary between the compounds; however, synergistic antifungal activity was observed when the compounds were combined in assays. Similarly, lobocyclamides A and B (from *Lyngbya confervoides*) synergistically inhibited the growth of *C. albicans* and *C. glabrata* in disc diffusion assays, while compounds A–C all showed moderate activity against these fungal strains when used alone [143].

Whilst the studies summarised herein demonstrate the potential of cyanobacterial-derived compounds in treating fungal infections, they also highlight the need for further studies to further evaluate the therapeutic potential of these compounds. In particular, several of the previous studies tested one (or limited) concentrations of the compounds and have reported potent activity on the basis of those evaluations. These studies need to be repeated with the activity screened across a range of concentrations and MICs reported. This would allow the activity of these compounds to be benchmarked against other antifungal compounds. Additionally, several of the studies that tested antifungal activity did not test toxicity in parallel, making it difficult to evaluate the safety of the compounds. 

### 4.3. Antiprotozoal Activity

Many compounds that have been isolated from cyanobacteria that inhibit bacterial growth are also effective at inhibiting some protozoal pathogens. Of these, the most studied protozoal organism is *P. falciparum*, the etiological agent of malaria tropica, which causes more than 600,000 deaths annually. Numerous drug-resistant strains have also emerged. However, the growth of *P. falciparum* and other protozoa can be inhibited by molecules derived from cyanobacteria. Shao et al. [105] reported IC_50_ values of 80–270 nM for bastimolides A and B against *P. falciparum*, alongside modest mammalian cell line toxicities of 2–3 µM, indicating their potential as anti-malarial chemotherapies. Similar findings were observed for calothrixin A and B [110], and for lagunamides A, B and C [89]. Additionally, carmabin is a highly effective inhibitor (MIC = 4.3 µM) of chloroquine-resistant *P. falciparum* [43]. Interestingly, dragonamide A and B do not inhibit *P. falciparum* or another parasite, *Trypanosoma cruzi*, but are effective inhibitors of *Leishmania donovani*, indicating that they may affect targets or processes that are specific to those protozoa [43,44]. Dragomabin, herbamide B and malyngolide dimer from *Lyngbya majuscula* also exhibit good antiparasitic activities of 4–20 µM against chloroquine-resistant *P. falciparum* [43,150].

The dudawalamide compounds (A–E) are good inhibitors *of P. falciparum*, *T. cruzi* and *L. donovani* growth, with MIC values ranging from 2.6 to 10 µM against all three parasites [126]. *Trypanosoma brucei brucei* is potently inhibited by both hoshinolactam and ikoamide, both of which produce MIC values in the nanomolar range [133,134], and can inhibit both the blood stage (*P. falciparum*) and liver stage (*P. berghei*) of *Plasmodium* spp., with lyngbyabellin A being the most potent inhibitor of both phases [136]. Phycocyanin, which was isolated from *Nostoc muscorum*, inhibited *P. falciparum* growth almost completely at 74 µg/mL [172]. The venturamides (A and B) inhibit *P. falciparum* growth [65], whilst the viridamides (A and B) inhibit *T. cruzi* and *L. mexicana* growth [46]. 

Marine cyanobacteria extracts containing compounds showing similarity to dioxanes, endoperoxides and spirocarbocyclic and spirooxindole substructures and have been also shown to inhibit the growth of *Leishmania infantum*, *Giardia duodenalis* and *Trichomonas vaginalis* parasites as adjudged by growth curve determinations [196], although IC_50_ values were not determined in that study. However, there are very few cyanobacterial compounds that have been examined for their antiparasitic activity against these protozoans. An exception is carmaphycin-17 (an analog derived from carmaphycin B from *Symploca cyanobacteria*), which has been shown to selectively inhibit the *T. vaginalis* 20S proteasome [197]. The proliferation of another parasite, *Encephalitozoon cuniculi*, has been reported to be inhibited by up to 50% by sulphated polysaccharides that were isolated from the cyanobacteria *Arthrospira platensis* [198,199].

### 4.4. Antiviral Activity

Substantially fewer studies have examined the antiviral activities of cyanobacterial compounds against viral pathogens than against the other classes of pathogens. Several reports have highlighted the potential of cyanobacterial compounds to treat viral diseases, although the range of viruses screened remains relatively narrow. Interestingly, antiviral activity has been most extensively studied against human immunodeficiency virus (HIV-1). Noteworthy anti-HIV activity was reported for the sulphated polysaccharide calcium spirulan, which was isolated from the marine cyanobacterium *Spirulina platensis* [112]. Indeed, an IC_50_ value of 9.3 μg/mL was determined in that study by determining viral release assays. This activity compared favourably to that of the dextran sulphate positive control (9.6 μg/mL) in that study. The same study also examined the effects of calcium spirulan on CD4 viability in HIV-infected cells, and the effects on HIV-induced syncytium formation, with noteworthy effects reported for all assays. Unfortunately, the toxicity of calcium spirulan was not evaluated in the same study, making it impossible to determine a therapeutic index (TI). However, a different study reported LC_50_ values of 2900–7900 μg/mL against several eukaryotic cell lines [113], indicating a therapeutic index of 300–800. At these high TIs, it is likely that calcium spirulan would be safe to use at therapeutic doses, although these studies should be repeated to test the antiviral activities and toxicity in parallel, within the same study. Notably, the Rechter et al. study [113] also screened calcium spirulan against human cytomegalovirus (HCMV), poliovirus and herpes simplex virus (HSV) and reported noteworthy activity (IC_50_ values 0.92–23 μg/mL), indicating that this compound is effective against a broad spectrum of viral pathogens. Further studies are required to evaluate the effects of calcium spirulan against other viruses.

Interestingly, several other sulphur-containing cyanobacterial compounds also have good anti-HIV activity. In one study, an uncharacterised sulpholipid fraction isolated from *Lyngbya lagerhimii* and *Phormidium tenue* had noteworthy anti-HIV activity between 1 and 100 μg/mL [188]. However, IC_50_ values were not reported in that study, making it difficult to compare the activity with other studies. Furthermore, that study did not screen these lipids for toxicity. Future studies are therefore required to evaluate its safety for therapeutic use. A different study reported similar antiviral activity for a similar sulfoglycolipid against HIV, with IC_50_ values as low as 24 nM [189]. The authors also reported that study determined that this compound inhibited the activity of DNA polymerase and postulated that the anti-HIV activity may be due to inhibition of HIV reverse transcriptase, although this has yet to be confirmed.

Microvirin (isolated from *Microcystis aeruginosa*) was also a potent inhibitor of HIV replication, with IC_50_ values in the range 2–12 nM against both HIV-1 and HIV-2 [155,156]. Notably, microviron was reported to be nontoxic against MT-4 and MVN T cells in those studies at concentrations up to 7 μM. These results equate to Tis of approximately 1000, indicating the safety of this compound for therapeutic use in the treatment of HIV. Another study reported good anti-HIV activity for scytovirin (isolated from *Scytonema varium*) against several HIV strains, with IC_50_ values 0.3–22 nM [186]. Interestingly, other studies have also reported scytovirin to have good inhibitory activity against several other serious human viral pathogens, including Ebola virus, Marburg virus and hepatitis C virus, with IC_50_ values between approximately 3 and 96 nM [117,185,187]. Furthermore, the toxicity was also evaluated in those studies, with LC_50_ values > 400 nM, indicating its safety for therapeutic use. Similarly, cyanovirin (isolated from *Nostoc ellipsosporum*) was also evaluated against HIV and reported to inhibit HIV cell entry via binding to HIV gp120 protein [118]. Other studies have also reported cyanovirin to inhibit the replication of Ebola virus, Marburg virus, hepatitis C, influenza and parainfluenza, with IC_50_ values as low as 50 nM [117,119,120,121]. None of these studies screened this compound for toxicity. Further studies are therefore required to determine the safety of cyanovirin for therapeutic use.

The antiviral effects of several cyanobacterial compounds have also been screened against herpes simplex virus (HSV). Three bauerines (A–C) isolated from *Dichotrix baueriana* were reported to have noteworthy anti-HSV-2 activity (IC_50_ = 3 μg/mL) [106]. That study also evaluated toxicity in LoVo cells and reported an LC_50_ of 5 μg/mL. This equates to a TI of substantially less than 2, which indicates that this compound may not be safe for treatment of HSV-2. Similar results were reported for a novel lectin isolated from *Ocillatoria acuminate* and *Ocillatoria agarghii*, with IC_50_ values in the range 90–130 μg/mL [165,166]. However, the LC_50_ values of 107 and 254 μg/mL against Huh-7 and MCF-7 cells, respectively, indicate that this compound is toxic and highlight the need for further studies to evaluate its safety for therapeutic use. In contrast, substantially better therapeutic potential was reported for nostoflan (isolated from *Nostoc flagelliforme*) against both HSV-1 and HSV-2 (IC_50_ values 0.4–100 μg/mL) [162]. Additionally, the authors of that study reported nostoflan to be nontoxic, with LC_50_ values 5–10 mg/mL. Thus, nostoflan is likely to be a substantially better antiviral option for treating HSV infections, although in vivo studies are required to further evaluate is potential.

Whilst the antiviral studies reviewed herein highlight the potential of some cyanobacterial compounds as antiviral therapeutics, they also highlight the substantial amount of work required. The compounds tested for antiviral activity represent only a small percentage of the cyanobacterial compounds identified to date. Furthermore, these compounds have only been tested against a limited panel of viral pathogens, and with a few notable exceptions, the antiviral mechanism(s) remain to be determined. Additionally, all of these reports have screened the compounds using in vitro assays and therefore do not account for the bioavailability of the compounds. Where noteworthy antiviral activities and Tis have been determined, the efficacy should also be verified in in vivo systems.

## 5. Conclusions

The development of multiple-drug-resistant pathogens has highlighted the need to develop new antimicrobial therapies. Much of the antibiotic development pipeline of new antibiotics over the last century has relied on studies evaluating bacteria (particularly soil bacteria) and fungi for compounds with antibacterial activity. In contrast, cyanobacterial compounds have been relatively neglected as antimicrobial therapies and substantially more work is warranted. Multiple novel compounds with antibacterial, antifungal, antiprotozoal and antiviral activity have already been reported. However, substantially more work is required to thoroughly evaluate the potency of these compounds, and to evaluate their safety for therapeutic use. Additionally, many more cyanobacterial species and countless strains are yet to be evaluated for similar activities, and where relevant, the noteworthy compounds identified. This review summarises the compounds that have been evaluated and highlights gaps in the literature, with the aim of stimulating interest in this field and focusing future studies.

## Figures and Tables

**Table 1:** Cyanobacterial natural products.

Cyanobacterial Compound	Source	Biological Activity	MIC, IC_50,_ GIR or DIZ	Toxicity	References
Abietane diterpenes	*Microcoleous lacustris*	Antibacterial activity against *S. aureus*, *S. epidermidis*, *S. typhi*, *V. cholerae*, *B. cereus*, *B. subtilis*, *E. coli*, *K. pneumoniae*	MIC = 14–22 μg/mL ^a^	Not reported in this study.	[91]
Aeruginazole A	*Microcystis* spp.	Inhibition of *B. subtilis*, *S. aureus*, *S. epidermidis*. Ineffective against *E. coli*.	2.2 μM ^a^	Not reported in this study.	[92,93]
Ambigol A–C	*Fischerella ambigua*	Antibacterial activity against *B. megaterium*	8 mm ^f^ at 100 nM	Not reported in this study.	[94,95]
Ambiguine isonitriles	*Fischerella ambigua*	Antibacterial activity against *B. anthracis*, *M. tuberculosis*, *S. aureus*	MIC = 78 ng/mL–2.5 μg/mL ^a^	Moderately toxic. LC_50_ not provided.	[96,97,98]
		Antifungal activity against *C. albicans*	MIC = 1 μM ^a^		
Anaephene A–C	*Hormoscilla* spp.	Antibacterial activity against *S. aureus*, MRSA, *M. luteus*, *B. cereus*	MIC = 6.1–24 μg/mL ^a^	LC_50_ = 26–>100 μg/mL in HCT116 cells	[99,100]
Anyhdrohapaloxindole A	*Fiscerella muscicola* and *Westiellopsis* spp.	Antibacterial activity against *M. tuberculosis*, *M. smegmatis*, *S. aureus*, *E. coli*, *A. baumannii*	MIC = <0.6–100 μg/mL^a^	LC_50_ = <9.2–>100 μg/mL against Vero cells	[101]
		Antifungal activity against *C. albicans*	MIC = 0.7–100 μg/mL ^a^		
Antillatoxin B	*Moorea producens*	Antibacterial activity against *B. cereus*, *S*, *typhimurium* and *L. monocytogenes*	7.8–500 μg/mL ^a^. Generally most potent against *B. cereus*	Not reported in this study.	[102]
Balticidins	*Anabaena cylindrica*	Antifungal activity against *C. maltosa*	6–18 mm ^f^ at 6 nM	Cytotoxicity was not reported in this study.	[103]
Bastadin	*Anabaena basta*	Antibacterial activity. Species not defined.	Antibacterial activity confirmed but not quantified.	Not reported in this study.	[104]
Bastimolide A and B	*Okeania hirsuta*	Antimalarial activity against *P. falciparum*	80–270 nM ^b^	LC_50_ against Vero cells = 2.1 μM	[105]
Bauerines A–C	*Dichotrix baueriana*	Anti-HSV-2 activity	IC_50_ = 3 μg/mL ^b^	LC_50_ = 5 μg/mL in LoVo cells.	[106]
Bromoanaindolone	*Anabaena constricta*	Antibacterial activity against *B. cereus*	530 μM ^a^	Not reported in this study.	[107]
Brunsicamine A–C	*Tychonema* spp.	Antibacterial activity against *Mycobacteria* spp.	2.6–5.7 μM ^b^	Not reported in this study.	[108]
Calophycin	*Calothrix fusca*	Antifungal activity against *A. oryzae*,	13 mm ^f^ at 1 nM	Not reported in this study.	[109]
		*C. albicans*,	7 mm ^f^ at 1 nM		
		*P. notatum*,	12 mm ^f^ at 1 nM		
		*S. cerevisiae* and	12 mm ^f^ at 1 nM		
		*T. mentagrophytes*	15 mm ^f^ at 1 nM		
Calothrixin A and B	*Calothrix* spp.	Antiprotozoal activity against *P. falciparum*	IC_50_ = 58–180 nM ^b^	LC_50_ = 40 nM against HeLa cells	[110]
Carbamidocyclophane F	*Nostoc* spp. UIC10274	Antibacterial inhibition of *M. tuberculosis*, *A. baumannii*, *P. aeruginosa*, *S. aureus*, *S. pneumoniae* and *E. faecalis*	0.8–5.4 μM ^a^ (*M. tuberculosis*); >10 μM ^a^ against all other bacteria	LC_50_ = 0.5–0.7 μM against MDA-MB-435 and HT-29 human cancer cell lines.	[111]
		Antifungal activity against *C. albicans*	>10 μM ^a^		
Carbamidocyclophane G		Antibacterial inhibition of *M. tuberculosis*, *A. baumannii*, *P. aeruginosa*, *S. aureus*, *S. pneumoniae* and *E. faecalis*	1.8–10 μM ^a^ against *M. tuberculosis*; >10 μM ^a^ against all other bacteria		
		Antifungal activity against *C. albicans*	>10 μM ^a^		
Calcium spirulan	*Spirulina platensis*	Antiviral activity against HIV, HCMV, Poliovirus and Herpes virus. Also has predicted activity against SARS -CoV-2	IC_50_ = 0.92–23 μg/mL ^b^	Low toxicity (LC_50_ = 2900–7900 μg/mL).	[112,113]
Carmabin	*Lyngbya majuscula*	Antiparasitic against *P. falciparum*	4.3 μM ^b^ against chloroquin-resistant strains.	Moderate toxicity in Vero cells.	[43]
Carriebowlinol	Not specified	Antibacterial activity against *Vibrio* spp.	<1 μM ^a^	Not reported in this study.	[114]
		Antibacterial activity against *Fusarium* spp.	0.2 μM ^a^		
Comnostins A-E	*Nostoc commune*	Antibacterial activity against *B. cereus*	40–300 μM ^a^	Cytotoxic LC_50_ = 1 μM	[115]
		*E. coli*	10–80 μM ^a^		
		*S. epidermidis*	150–300 μM ^a^		
Crossbyanol A–C	*Leptlyngbya crossbyna*	Antibacterial activity against *S. aureus*	3 μM ^a^	Cytotoxic in brine shrimp assay (LC_50_ = 3 μM)	[116]
Cyanovirin	*Nostoc ellipsosporum*	Antiviral activity against HIV, SIV, Hepatitis C, Ebola virus, Parainfluenza virus (type 3), Influenza virus	IC_50_ = 50 nM (against Ebola and Marburg viruses) ^b^. It has been shown to block HIV cell entry by interacting with HIV gp120.	Not reported in these studies.	[117,118,119,120,121]
Cybastacines A and B	*Nostoc* spp.	Antibacterial activity against *E. faecalis*, *E. faecium*, *M. abscessus*, *N. carnea*, *N. cyriacigeorgica*, *S. pyogenes*, *S. aureus*, *S. epidermidis*	4–32 μg/mL ^b^	Not reported in this study.	[122]
Cylindrofridin A–C	*Cylindrospermum stagnale*, *Nostoc* spp.	Antibacterial activity against *S. aureus*, *MRSA*, *S. pneumoniae*, *E. faecium* and a wide panel of other bacteria	MIC = 1 μg/mL against *S. aereus*. ^a^ Substantially higher MICs against other bacterial species.	LC_50_ = 0.9–14 μg/mL against HaCaT cells.	[123]
Cylindrocyclophanes A–C					
Corioloc acid	*Oscillatoria redekei*	Antibacterial activity against *B. subtilis*, *M. flavus*, *S. aureus*, *S. epidermidis*	2–9 mm ^f^ at 50 μg/disc	Not reported in this study.	[124]
Dimorphecolic acid			3–8 mm ^f^ at 100 μg/disc		
Crossbyanols A–D	*Lyngbya* spp.	Antibacterial activity against *S. aureus*, MRSA	MIC = 2–4 μg/mL ^a^	LC_50_ = 30 μg/mL in brine shrimp toxicity assay.	[116]
Didehydromirabazole	*Scytonema mirabile*	Antifungal activity against *A. oryzae*, *C. albicans*, *P. notatum*, *S. cerevisiae*, *T. mentagrophytes*	8–34 mm ^f^	LC_50_ = 0.5–99 μg/mL against KB cells	[125]
Dragonamide A	*Lyngbya majuscula*	Antiparasitic against *P. falciparum*, *T. cruzi*, *L. donovani*	5.1 μM ^c^ against *L. donovani*; ineffective against *P. falciparum* and *T. cruzi*	Not reported.	[43,44]
Dragonamide E			6.5 μM ^c^ against *L. donovani*; ineffective against *P. falciparum* and *T. cruzi*		
Dragomabin	*Lyngbya majuscula*	Antiparasitic against *P. falciparum*	1.4–21 μM ^b^ against chloroquin-resistant strains.	Moderate toxicity with LC_50_ = 182 μM in Vero cells. A therapeutic index of >30, indicating its therapeutic safety.	[43]
Dudawalamide A	*Moorea producens*	Antiparasitic against *P. falciparum*, *T. cruzi*, *L. donovani*	2.6–10 μM ^a^	Nontoxic at 30 μM against H-460 human lung cancer cells.	[126]
Dudawalamide B			6–10% ^a^		
Dudawalamide C			10 μM ^a^		
Dudawalamide D			10 μM ^a^		
Dudawalamide E			10 μM ^a^		
Eucapsitrione	*Eucapsis* spp.	Antibacterial and antifungal activity against *M. tuberculosis*, *M. smegmatis*, *S. aureus*, *E. coli*, *C. albicans*	MIC = 3.1–6.4 μM against M. tuberculosis ^a^. Substantially less active against other bacteria.	IC_50_ > 20 μM in Vero cells	[127]
Fischerellin A and B	*Fischerella muscicola*	Antibacterial activity against *S. aureus*, *M. tuberculosis*, *M. smegmatis*, *E. coli* and antifungal activity against *C. albicans*	MIC = 2–100 μM ^a^	LC_50_ = >128 μM in Vero cells	[97]
		Antifungal activity against *U. appendiculatus* and *E. grammis*	100% inhibition between 0.6 and 2.5 mM	Not reported in this study.	[128]
Fischambiguine B	*Fischerella ambigua*	Antibacterial activity against *S. aureus*, *B. anthracis*, *M. tuberculosis*, *M. smegmatis*	MIC = 2–100 μM ^a^	LC_50_ = >128 μM in Vero cells	[97]
Hapalindole (multiple)	*Nostoc* spp. *Fischerella* spp.	Antibacterial activity against *S. aureus*, *B. subtilis*, *E. coli*, *E. faecium*, *S. epidermidis*, *S. pyogenes*, *S. pneumoniae*, *H. influenza*, *K. pneumoniae*, *P. morganii*, *Salmonella* spp., *S. sonnei*	30–36 mm ^f^	Moderate toxicity (LC_50_ >30 μM)	[129,130]
	*Hapalosiphon fontinalis*	Antifungal activity against *C. albicans*	0.7 μM ^a^	LC_50_ = 12–44 μM	[101]
Hassallidins A and B	*Hassallia* spp.	Antifungal activity against *A. fumigatus* and *C. albicans*	3.5 μM ^a^	Not reported in this study.	[131,132]
Herbamide B	*Lyngbya majuscula*	Antiparasitic against *P. falciparum*	5.9 μM ^b^ against chloroquin-resistant strains.	Moderate toxicity in Vero cells.	[43]
Hoshinolactam		Antiparasitic activity against *T. brucei brucei*	3.9 nM ^b^	Not reported in this study.	[133]
Ikoamide	*Okeania* spp.	Antiparasitic against *P. falciparum*	0.14 μM ^b^	Not reported in this study.	[134]
Ichthyopeptins	*Microcoleous ichythyoblabe*	Antiviral activity against influenza A virus. Additionally, predicted anti-SARS-CoV-2 activity due to ACE inhibitory activity.	IC_50_ = 12.5 μg/mL ^b^	Not reported in this study.	[135]
Kakeromide B	*Moorea producens*	Antimalarial activity against *P. falciparum* (blood stage) and *P. berghei* (liver stage)	0.9–1.2 μM ^b^	LC_50_ against HEK293T and HepG2 cells = >2.3 μM	[136]
Kawaguchipeptins A and B	*Microcystis aeruginosa*	Antibacterial activity against *S. aureus*	0.7 mM ^a^	Not reported in this study.	[137]
Lagunamide A–C	*Lyngbya majuscula*	Inhibition of *P. aeruginosa* swarming	Inhibited 49% of swarming ^e^	Cytotoxicity against P388 murine leukaemia, A549 human lung carcinoma, PC3 (human prostate cancer), HCT8 (colorectal adenocarcinoma) and SK-OV (ovarian cancer) cell lines with LC_50_ values 2.1–4.5 nM.	[89,138]
		Antiparasitic against *P. falciparum*	0.29 μM ^b^		
Laxaphycin A	*Moorea producens*	Antibacterial activity against *B. cereus*, *S. typhimurium* and *L. monocytogenes*	150–500 μg/mL ^a^.	Not reported in this study.	[102]
Laxaphycin B					
Laxaphycin B3					
Laxaphycins (several)	*Anabaena laxa*, *Moorea producens*, *Anabaena torulosa*	Antifungal activity against *A. oryzae*	20 μM ^a^	LC_50_ = 0.2 μM	[63,139,140,141]
Linoleic acid	*Oscillatoria redekei*	Antibacterial activity against *B. subtilis*, *Micrococcus flavus*, *S. aureus*, *S. epidermidis*	2–18 mm ^f^ at 100 μg/disc	Not reported in this study.	[124]
ϒ-Linolenic acid	*Fischerella* spp.	Antibacterial activity against *S. aureus*, *E. coli*, *K. aerogenes*, *P. aeruginosa*, *S. typhi*	12–22 mm ^f^; MIC = 2–16 μg/mL ^a^	Not reported in this study.	[142]
Lobocyclamide A–D	*Lyngbya confervoides*	Antifungal activity against *C. albicans*	10 μM ^a^	Not reported in this study.	[143]
Lyngbic acid	*Moorea producens*	Antibacterial inhibition of *Mycobactetium tuberculosis*	65% ^d^	LC_50_ in MDA-MB-231, A549 and HT-29 cells = 0.05–88 μM.	[144]
Lyngbyabellin A and B, 18E-lyngbyalosise C, lyngbyaloside	*Moorea producens*	Antimalarial activity against *P. falciparum* (blood stage) and *P*. *berghei* (liver stage)	0.15 nM–>4 μM ^b^. Lyngbyallin A was ~1000-fold more potent than the other compounds.	LC_50_ against HEK293T and HepG2 cells = 0.3–>4.8 μM	[136]
Lyngbyabellin B	*Lyngbya majuscula*	Antifungal activity against *C. albicans*	10.6 mm ^f^	Potent toxicity (LC_50_ = 3 ppm) in *Artemia* nauplii toxicity assay.	[145]
Lyngbyazothrins	*Lyngbya* spp.	Antibacterial activity against *B. subtilis*, *E. coli*	18 mm ^f^ at 16–65 μM	Not reported in this study.	[146,147,148]
Malyngamide 4	*Moorea producens*	Antibacterial inhibition of *M. tuberculosis*	10–18% ^d^	LC_50_ in MDA-MB-231, A549 and HT-29 cells = 0.05–88 μM.	[144]
Malyngamide A					
Malyngamide B					
Malyngoloide	*Lyngbya masculata*	Antibacterial activity against *B. cereus*, *S. aureus* and *S. pyogenes*	Reported but not quantified.	Not reported in that study.	[149]
Malyngolide dimer	*Lyngbya majuscula*	Antiparasitic against *P. falciparum*	19 μM ^b^ against a chloroquin-resistant strain	Not reported in that study.	[150]
Majusculamide A	*Moorea producens*	Antibacterial activity against *B. cereus*, *S. typhimurium* and *L. monocytogenes*	63–>500 μg/mL ^a^. Generally most potent against *B. cereus*	Not reported in this study.	[102]
Majusculamide C					
Majusculamide C acetate					
Majusculamide I					
Majusculamide J					
Majusculamide C	Not specified	Antifungal activity against *R. solani*, *P. aphanidermatum*, *A. euteiches*, *P. infestans*	<1–4 μM ^a^	LC_50_ = 20–750 nM	[151,152,153]
Microcystin-LR	*Microcystis* spp.	Antibacterial activity against *M. chelonae*, *M. kansaii*, *M. terrae*, *M. tuberculosis*	MIC = 60 nM–1.93 μM ^a^	Nontoxic to HTC cells.	[154]
Microvirin	*Microcystis aeruginosa*	Antiviral activity against HIV-1 and HIV-2. Inhibits virus–cell fusion.	2–12 nM ^b^	Nontoxic in MT-4 and MVN T cells at concentrations ≤7 μM	[155,156]
Muscoride	*Nostoc muscorum*	Antibacterial activity against *B. subtilis*	3–6 mm ^f^	Not reported in this study.	[157]
20-Nor-3α-acetoxy-abieta-5,7,9,11,13-pentaene	*Microcoleous lacustris*	Antibacterial activity against *S. aureus*, *S. epidermidis*, *S. typhi*, *V. cholerae*, *B. subtilis*, *B. cereus*, *E. coli*, *K. pneumoniae*	14–286 μg/mL ^a^. These diterpenoids were most potent against *Staphylococcus* spp.	Not reported in this study.	[91]
20-Nor-3α-acetoxy-12 hydroxy-abieta-5,7,9,11,13-pentaene					
Norharmane (9H-pyrido(3,4-*b*)indole	*Nodularia harveyana* and *Nostoc insulare*	Antibacterial activity against *E. coli*, *P. aeruginosa*, *S. aureus*, *B. subtilis*, *B. cereus*	MIC values of 32 μg/mL (*E. coli* and *P. aeruginosa*), 160 μg/mL (*B. cereus*), 128 μg/mL (*B. subtilis*) and 16 μg/mL (*S. aureus*) ^a^	Not reported in this study.	[158]
		Antifungal activity against *C. albicans*	40 μg/mL ^a^		
4,4’-Dihydroxybiphenol		Antibacterial activity against *E. coli*, *P. aeruginosa*, *S. aureus*, *B. subtilis*, *B. cereus*	MIC values of >128 μg/mL (*E. coli* and *P. aeruginosa*), 32 μg/mL (*B. cereus*), 128 μg/mL (*B. subtilis* and *S. aureus*) ^a^		
		Antifungal activity against *C. albicans*	32 μg/mL ^a^		
Noscomin	*Nostoc commune*	Antibacterial activity against *B. cereus*, *S. epidermidis*, *E. coli*	18–300 μM ^a^	Not reported in this study.	[159]
Nostofungicidine	*Nostoc commune*	Antifungal activity against *A. candidus*	1.5 μM ^a^	LC_50_ = 1.5 μM against NSF-60 cells	[160]
Nostocionone	*Nostoc commune*	Antibacterial activity against *P. acnes*	~10 mm at 50 μg/disc ^f^	Not reported in this study.	[161]
Nostocionone D1			~8mm at 50 μg/disc ^f^		
Nostocionone D2			~9 mm at 50 μg/disc ^f^		
Nostocionone D3			~11.5 mm at 50 μg/disc ^f^		
Nostoflan	*Nostoc flagelliforme*	Antiviral activity against HSV-, HSV-2, HCMV, influenza, adenovirus, coxsackie virus	IC_50_ = 0.37–100 μg/mL. ^b^ Particularly good against HSV-1.	Nontoxic. LC_50_ = 4.9–>10 mg/mL	[162]
Nostocyclyne A	*Nostoc* spp.	Antibacterial activity against *S. aureus*, *B. subtilis*	30–36 nM ^a^	Not reported in this study.	[163]
Nostotrebin 7 and nostolactone 7	*Nostoc* spp.	Antibacterial activity against *E. faecium*, *B. subtilis*, *S. aureus*, *M. tuberculosis*, *E. aerogenes*, *S. typhi*, *P. aeruginosa*, *E. coli*	MIC = 2–16 μg/mL ^a^	Not reported in this study.	[129,164]
Novel *Oscillatoria* lectin	*Ocillatoria acuminate*, *Ocillatoria agarghii*	Antiviral activity against HSV-1	IC_50_ = 91–131 μg/mL. ^b^	EC_50_ = 107 (Huh-7 cells) and 254 μg/mL (MCF-7 cells).	[165,166]
Pahayokolide A and B	*Lyngbya* spp.	Antibacterial activity against *B. subtilis*, *B. megaterium*, *P. aeruginosa*, *M. luteus*, *E. coli* and *S. epidermidis*	Only inhibited *Bacillus* spp. with 32 mm at 5 μg/mL ^f^	Acute toxicity in zebrafish assay (100% mortality at 3 μg/mL).	[167,168]
C-Phycocyanin	Multiple *Westiellopsis* spp. (specific species not identified)	Antibacterial activity against *B. subtilis*, *Pseudomonas* spp.; *Xanthamonas* spp.	1.3–13.2 mm ^f^. Generally most potent against *B. subtilis*	Nontoxic in silkworm toxicity assay.	[169]
Parsiguine	*Fischerella* spp.	Antibacterial activity against *S. epidermidis* and antifungal activity against *C. krusei*	MIC = 40 μg/mL (*S. epidermidis*) ^a^ and 20 μg/mL (*C. krusei*) ^a^	Not reported in this study.	[170]
Phycocyanin	*Spirulina* spp.	Antibacterial activity against *S. aureus*, *E. coli*, *P. aeruginosa*, *K. pneumoniae*	MIC = 50–125 μg/mL ^a^	Not reported in this study.	[171]
	*Nostoc muscorum*	Antiparasitic activity against *P. falciparum*	95% inhibition at 74 μg/mL	Not reported in this study.	[172]
	*Nostoc* spp.	Antibacterial activity against *S. aureus*, *Pseudomonas* spp. *Klebsiella* spp.	5–13 mm ^f^	Not reported in this study.	[173]
Pitipeptolide A	*Lyngbya majuscula*	Antibacterial. Inhibition of *M. tuberculosis*	9–30 mm ^f^ against several *M. tuberculosis* strains.	Weak cytotoxicity against Vero cells (LC_50_ = 2–2.25 μg/mL)	[174]
Pitipeptolide B					
Pitiprolamide	*Lyngbya* spp.	Antibacterial activity against *B. cereus*, *M. tuberculosis*	10–40 mm at 100 μg in disc ^f^	Weak toxicity (Lc50 = 11–>100 μM) in HT-29 and MCF7 cells	[175]
Plastimolide A	Not specified	Antiprotozoal activity against *P. falciparum*, *L. donovani*	173 nM (*P. falciparum*), 4.7 μM (*L. donovani*) ^b^	Low toxicity in GepG2 cells (LC_50_ = 5 μM)	[176]
Protoamides	*Photrmidium* spp.	Antibacterial activity against *M. luteus*, *B. subtilis*, *E. coli*	Antibacterial reported but not quantified.	Not reported in this study.	[94]
Schizotrin A	*Schizotrix* spp.	Antibacterial activity against *B. subtilis*	15 mm ^f^ at 7 nM	Not reported in this study.	[177]
Scytophytins and tolytoxins	*Scytonema* spp. and *Tolypothrix* spp.	Antifungal activity against *S. pastorianus*, *N. crassa*, *C. albicans*, *P. ultimum*, *R*, *solani*, *S. homoeocarpa*	24–30 mm at 1.2 μM ^f^	LC_50_ = 50–100 nM	[178,179,180,181,182,183]
Scytoscalarol	*Scytonema* spp.	Antibacterial activity against *B. anthracis*, *S. aureus*, *E. coli*, *M. tuberculosis*	2–110 μM ^a^	Weak cytotoxicity (LC_50_ = 135 μM)	[184]
		Antifungal activity against *C. albicans*	4 μM ^a^		
Scytovirin	*Scytonema varium*	Antiviral activity against HIV, Ebola virus (Zaire strain), Marburg virus, Hepatitis C	IC_50_ = 0.3–22 nM (HIV strains), 41 nM (Zaire Ebola virus), 3.2–96 nM (Marburg virus and Hepatitus C virus). ^b^	LC_50_ > 400 nM	[117,185,186,187]
Sulfolipids	*Lyngbya lagerhimii*, *Phormidium tenue*	Antiviral activity against HIV	Inhibitory at concentrations between 1–100 μg/mL, although no IC_50_ is recorded.	Not reported in this study.	[188]
Sulfoglycolipid	*Scytonema* spp.	Antiviral activity against HIV. Activity against viral reverse transcriptase (recorded as DNA polymerase in that study).	IC_50_ = 24 nM–100 μM ^b^	Not reported in this study.	[189]
Tanikolide	*Scytonema* spp.	Antifungal activity against *C. albicans*	13 mm at 350 nM ^f^	LC_50_ = 12–32 μM	[190,191]
Tiahuramide A	*Lyngbya majuscula*	Antibacterial activity against *S. baltica*, *A. salmonicida*, *V. anguillarum*, *M. luteus and E. coli.*	6.7–47 μM ^a^	LC_50_ values 6–14 μM against SH-SY5Y human neuroblastoma cells	[192]
Tiahuramide B					
Tiahuramide C					
Tjipanazole D	*Fischerella* spp.	Antibacterial activity against *M. luteus*, *B. subtilis*, *E. coli*	Antibacterial reported but not quantified.	Not reported in this study.	[193]
Tolybyssidins A and B	*Tolypothrix byssoides*	Antifungal activity against *C. albicans*	22 and 42 μM ^a^	Not reported in this study.	[194]
Ulongamide A	*Moorea producens*	Antimalarial activity against *P. falciparum* (blood stage) and *P. berghei* (liver stage)	1–4 μM ^b^	LC_50_ against HEK293T and HepG2 cells > 2.3 μM	[136]
Venturamides A and B	*Oscillatoria* spp.	Antiprotozoal activity against *P. falciparum*	5.6 μM ^b^	Mild cytotoxicity (LC_50_ = 86 μM) against Vero cells	[65]
Viridamides A and B	*Oscillatoria nigro-viridis*	Antiprotozoal activity against *T. cruzi* and *L. mexicana*	1.1–1.5 μM ^b^	Not reported in this study.	[46]

^a^ minimum inhibitory concentration (MIC); ^b^ IC_50_ = 50% inhibitory concentration; ^c^ growth inhibitory rate at 10 μg/mL; ^d^ growth inhibitory rate at 12.5 μg/mL; ^e^ growth inhibitory rate at 100 ppm; ^f^ diameters of inhibition zone (mm).

## Data Availability

All data are available from the corresponding author on reasonable request.
